# Modelling myocardial ischemia/reperfusion injury with inflammatory response in human ventricular cardiac organoids

**DOI:** 10.1111/cpr.13762

**Published:** 2024-10-08

**Authors:** Laihai Zhang, Yun Jiang, Wenwen Jia, Wenjun Le, Jie Liu, Peng Zhang, Huangtian Yang, Zhongmin Liu, Yang Liu

**Affiliations:** ^1^ Shanghai Heart Failure Research Center, Shanghai East Hospital, School of Medicine Tongji University Shanghai China; ^2^ Department of Cardiovascular Surgery, Shanghai East Hospital, School of Medicine Tongji University Shanghai China; ^3^ Institute for Regenerative Medicine, Shanghai East Hospital, School of Medicine Tongji University Shanghai China; ^4^ National Stem Cell Translational Resource Center, Shanghai East Hospital, School of Life Sciences and Technology Tongji University Shanghai China; ^5^ Laboratory of Molecular Cardiology, Shanghai Institute of Nutrition and Health University of Chinese Academy of Sciences (CAS) Shanghai China; ^6^ Shanghai Institute of Stem Cell Research and Clinical Translation Shanghai China

## Abstract

Current therapeutic drug exploring targeting at myocardial ischemia/reperfusion (I/R) injury is limited due to the lack of humanized cardiac models that resemble myocardial damage and inflammatory response. Herein, we develop ventricular cardiac organoids from human induced pluripotent stem cells (hiPSCs) and simulate I/R injury by hypoxia/reoxygenation (H/R), which results in increased cardiomyocytes apoptosis, elevated oxidative stress, disrupted morphological structure and decreased beat amplitude. RNA‐seq reveals a potential role of type I interferon (IFN‐I) in this I/R injury model. We then introduce THP‐1 cells and reveal inflammatory responses between monocytes/macrophages and H/R‐induced ventricular cardiac organoids. Furthermore, we demonstrate Anifrolumab, an FDA approved antagonist of IFN‐I receptor, effectively decreases IFN‐I secretion and related gene expression, attenuates H/R‐induced inflammation and oxidative stress in the co‐culture system. This study advances the modelling of myocardial I/R injury with inflammatory response in human cardiac organoids, which provides a reliable platform for preclinical study and drug screening.

## Graphical abstract

We obtained human ventricular cardiac organoids from hiPSCs by inhibiting the retinoic acid signaling pathway in self‐organizing differentiation and co‐cultured them with monocytes under hypoxia/reoxygenation to model myocardial ischemia/reperfusion injury. Through RNA‐seq we found that IFN‐I may be a potential therapeutic target and verified the efficacy of related drug Anifrolumab.

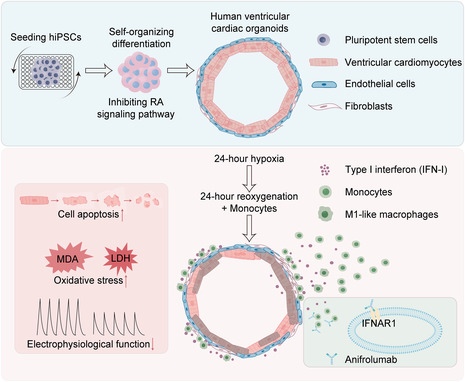

## 1 INTRODUCTION

Acute myocardial infarction is a severe myocardial injury affecting over 7 million people worldwide each year.^1^ The most effective clinical practice for myocardial recovery is timely reperfusion. However, reperfusion also causes a series of pathophysiological responses known as ‘ischemia/reperfusion (I/R) injury’,^2^ which accounts for nearly 50% of the final infarct size.^3^ Currently, available drugs targeting at the myocardial I/R injury are limited. A number of large‐scale clinical trials have been conducted with the aim to alleviate reperfusion injury, such as the cyclosporine injection or ischemic postconditioning, while all of these strategies failed to rescue adverse clinical outcomes though effective in animal studies.^4^, ^5^, ^6^ One of the causes is that knowledges and exploring protective drugs related to myocardial I/R injury are mainly based on the animal studies due to the lack of humanized myocardial models.

More than 80% of drugs exhibited different physiological effects between human and animal models.^7^ A major obstacle is the substantial disparity of cells for animals and humans in terms of genetics and physiology.^8^ For instance, there are clear distinctions in beating frequency, oxygen consumption, myofilament structure and ion channels of cardiomyocytes (CMs).^9^ To date, most of the reported myocardial I/R injury models are based on surgery or drugs on rodent animals.^10^ Hence, developing humanized myocardial I/R models based on human‐derived cardiac cells to precisely resemble responses of the human ventricular tissue to I/R insult and to protective reagents are urgently needed to overcome the wide gap between the mechanism and drug screening research and clinical studies.

Over the past decade, monolayer‐based differentiation of human pluripotent stem cells (hPSCs), especially human induced pluripotent stem cells (hiPSCs) to CMs using small‐molecules has become a well‐established research approach, mitigating issues related to genetic background.^11^ But some limitations, such as loss of cellular diversity and shortage of physiologically relevant architecture, still exist.^12^ Cardiac organoids have been developed that mimic the cell type, morphological structure and electrophysiology of the human heart. Recent studies have produced self‐organizing cardiac organoids with myocardial cavities from hPSCs,^13^, ^14^ which allows the coexistence of CMs, endothelial cells (ECs), and fibroblasts in a certain proportion. On the other hand, the newly developed chamber‐like structure can address the natural hypoxia for the innermost cells of solid‐type organoids.^15^, ^16^ These cardiac organoids replicated some native characteristics of the heart, making it closer to the real in vivo environment, holding the potential to ultimately replace monolayer CMs‐based experiments and animal studies.^17^ However, whether these cardiac organoids can well simulate myocardial I/R injury and have proper inflammatory responses between CMs, ECs and immune cells remain unclear.

Herein, we have successfully established a differentiation protocol for the generation of human ventricular cardiac organoids by inhibiting the retinoic acid (RA) signalling pathway during cardiac mesodermal stage. Then, we established simulated I/R injury model by taking these organoids under hypoxia/reoxygenation (H/R) stimulation and revealed the characteristics of the injury in terms of cell apoptosis, oxidative stress, disrupted morphological structure and aberrated electrophysiological function. Through analysis of transcriptome data, we identified the potential role of type I interferon (IFN‐I) related genes in promoting inflammation. Subsequently, Tohoku Hospital Paediatrics‐1 (THP‐1) immune cells were introduced into the culture system and the inflammatory response between H/R‐induced ventricular cardiac organoids and monocytes/macrophages was observed. Finally, the potential therapeutic efficacy of Anifrolumab, an IFN‐I receptor antagonist, was validated by showing the attenuation of inflammatory response and alleviation of H/R‐induced oxidative stress. Our findings demonstrate that humanized ventricular cardiac organoids can be a reliable alternative model for preclinical study of myocardial I/R injury and for therapeutic drug screening.

## 2 METHODS

2.1

2.1.1

This study did not generate new unique reagents.

### 2.2 hPSCs culture

The hiPSCs line ZB11AOF was sourced from biobank of Shanghai East Hospital, and the *MYL2‐Venus* human embryonic stem cells (hESCs) reporter line was sourced from Shanghai Institute of Nutrition and Health.^18^ hPSCs were nurtured on human‐qualified Matrigel (BD Biosciences) within the mTeSR1 medium (STEMCELL Technologies) to facilitate feeder‐independent and monolayer‐based growth. Tests for mycoplasma contamination were conducted regularly.

### 2.3 Cardiac organoid formation

On Day 1, hPSCs were detached into individual cells by Accutase (Thermo Fisher Scientific) and seeded at a density of 5000 cells in 200 μL mTeSR1 medium per well into ultra‐low‐attachment 96‐well plates (Corning) with 10 μM Y‐27632 (Selleck Chemicals). The plates were then centrifuged for 5 min at 200 g to aggregate the cells.^13^ On Day 0, cells formed spherical structures and were subsequently induced for 2 days using chemically defined medium (CDM) (Each 1000 mL consisted of 10 mL Insulin‐Transferrin‐Selenium, 10 mL GlutaMAX, 10 mL concentrated lipids, 10 mL Pen Strep, 40 μL monothioglycerol, 60 mL of a 150 mg/mL BSA solution, and 900 mL of a 1:1 mixture of F12 and IMDM) supplemented with FGF2 (50 ng/mL, Abclonal), BMP4 (10 ng/mL, Abclonal), Activin A (50 ng/mL, Abclonal), LY294002 (5 μM, Selleck Chemicals), CHIR99021 (8 μM, Selleck Chemicals) and Insulin (1 μg/mL, Beyotime). On Day 2, the medium was replaced to CDM medium supplemented with FGF2 (10 ng/mL), BMP4 (15 ng/mL), IWP2 (5 μM, Selleck Chemicals), VEGF‐A (200 ng/mL, Abclonal), RA (0.5 μM, Selleck Chemicals) or BMS493 (0.5 μM, MedChemExpress), and insulin (10 μg/mL) for 4 days. Medium was changed every 2 days. On Day 6, the medium was replaced to CDM medium supplemented with FGF2 (10 ng/mL), BMP4 (20 ng/mL), VEGF‐A (100 ng/mL) and insulin (10 μg/mL) for 2 days. After Day 8, the basal medium was replaced with DMEM/F12 containing 10% serum and 1% Pen Strep. Medium was changed every 3 days until Day 21.

### 2.4 H/R modelling and THP‐1 cells co‐culture

On Day 21, the medium of ventricular cardiac organoids were replaced to DMEM/F12 without glucose or serum and switched to incubator with 37°C, 5% CO_2_ and 0.1% O_2_. After 24 h, ventricular cardiac organoids were transferred back to incubator with 37°C, 5% CO_2_ and 21% O_2_ and the medium was retrieved with DMEM/F12 containing glucose and 10% serum. THP‐1 cells were re‐suspended at a density of 1 × 10^5^ cells per mL in DMEM/F12 containing 10% serum. For direct co‐culture, 200 μL of THP‐1 cell suspension as medium were placed into per well of one ventricular cardiac organoid. For indirect co‐culture, THP‐1 cell suspension and the organoids were separated by the transwell insert. To inhibit the activation of IFN‐I, the human monoclonal antibody Anifrolumab (MedChemExpress, HY‐P99168) was added during the reoxygenation phase.

### 2.5 Quantitative real‐time PCR

Total RNA was isolated and purified by EZ‐press RNA Purification Kit (EZBioscience). Subsequently, 500 ng RNA was reverse transcribed into complementary DNA (cDNA) using the RT Kit (with gDNA Remover) (EZBioscience) and set as the template for the 10 μL reaction system with SYBR Green (EZBioscience) MasterMix and forward/reverse primers. Quantitative real‐time PCR was performed by Quant‐Studio 7 Flex (Applied Biosystems). The relative expression was calculated using the 2^−ΔΔCt^ method and normalized by GAPDH expression. All the primers were listed in Table S1.

### 2.6 Flow cytometry assays

Ventricular cardiac organoids or THP‐1 cells were dissociated into single‐cell suspensions using a digestion solution consisting of a 1:1 mixture of Accutase and 0.25% trypsin. Protocol of flow cytometry assays was provided by Biolegend (https://www.biolegend.com/). In short, after washing by Cell Staining Buffer (Biolegend, 420,201), cells were fixed by Fixation Buffer (Biolegend, 420,801) for 20 min and washed with Intracellular Staining Perm Wash Buffer (Biolegend, 421,002) three times. For CMs of ventricular cardiac organoids, single‐cell suspensions were incubated with Cardiac Troponin T antibody (Abcam, ab45932) diluted in Intracellular Staining Perm Wash Buffer for 1 h at room temperature, washed twice with Wash Buffer, centrifuged, and incubated with secondary antibody in dark for 30 min at room temperature, shedding from light.^19^ For THP‐1 cells, human antibodies against CD86 (PE anti‐human CD86, Biolegend, 374,205) and CD206 (APC anti‐human CD206, Biolegend, 321,109) were added in, and staining was conducted in the reaction volume of 100 μL for 30 min at room temperature, shedding from light. For the MLC2v‐Venus positive cells of cardiac organoids, flow cytometry was performed immediately after fixation with 1% paraformaldehyde on the single‐cell suspension. For phagocytic function staining of THP‐1 cells, single‐cell suspensions were incubated with pHrodo Detection Kit (Invitrogen, P35372), following the manufacturer's protocol for staining. For apoptosis staining of ventricular cardiac organoids, single‐cell suspensions were incubated with Annexin V‐FITC/PI Apoptosis Detection Kit (Yeasen, 40302ES20), following the manufacturer's protocol for staining. Finally, after sufficient washing, marked cells were prepared for flow cytometry assays in 500 μL Cell Staining Buffer. Flow cytometry was conducted with a CytoFLEX S flow cytometer (Beckman Coulter, Brea, CA, USA) supported by CytExpert software (Version 2.4). Data were analysed by using FlowJo software (Version 10.8.1, Ashland, OR, USA).

### 2.7 Immunofluorescence (IF) staining

For TUNEL (TdT mediated dUTP Nick End Labeling) staining, ventricular cardiac organoids were collected and fixed at 4% (vol/vol) paraformaldehyde (Yeasen). Subsequently, the staining procedure was carried out according to protocol of TUNEL assay kit (Yeasen, 40307ES20). After completing the staining, the ventricular cardiac organoids were suspended in confocal dishes and images were captured under a Leica SP8 confocal microscope. For co‐culture staining of ventricular cardiac organoids and THP‐1 cells, ventricular cardiac organoids and THP‐1 cells were stained with DIO or PKH26 respectively before modelling.^20^ After the co‐culture, they were stained with DAPI together and taken image by confocal microscope.

For paraffin embedding and immunohistochemistry, ventricular cardiac organoids were embedded in paraffin after pre‐embedding in agarose. Sections around the largest cross‐section were used for IF staining. In brief, the sections underwent deparaffinization using xylene and ethanol gradients, followed by microwave antigen retrieval (Servicebio, G1207). Subsequently, permeabilization and block were performed (Beyotime, P0260), and primary antibodies were added and incubated overnight at 4°C. The primary antibodies were as follows: Cardiac Troponin T (Abcam, ab45932), CD31 (Abcam, ab24590), Vimentin (Abcam, ab24525), FAP (ABclonal, A23789), MYL2 (Synaptic System, 310,003) and MYL7 (Synaptic System, 311,011). Incubation of secondary antibodies and DAPI followed. Finally, confocal microscopy was used for taking image.

### 2.8 Malondialdehyde, lactate dehydrogenase and superoxide dismutase assay

The levels of superoxide dismutase (SOD), lactate dehydrogenase (LDH) and malondialdehyde (MDA) were tested with commercially available kits (Beyotime Biotechnology, S0101S/C0016/S0131S). In brief, after the collection of ventricular cardiac organoids, protein extraction was performed using cell lysis buffer (Beyotime Biotechnology, P0013), followed by quantification using BCA quantitative kit (Thermo Scientific, 23,225). The specific loading volume was then calculated. The culture supernatant was also collected after centrifugation to remove cell debris. Reagents were prepared according to the manufacturer's instructions and proceed with sample testing.

### 2.9 Multi‐electrode array test

The multi‐electrode array (MEA) plate was transferred to the MEA device, Maestro Edge version (Axion BioSystems Inc.; Atlanta, GA, USA) for measuring the spike amplitude and beat amplitude of field potential under spontaneous beating without electrical stimulation.

### 2.10 Elisa

Ventricular cardiac organoids culture supernatant was collected and stored at −80°C. Protein levels of IFNα (Absin, 551,025) and IFNβ (WEIAOBI, EH10229) were determined with specific ELISA kits according to the manufacturer's directions.

### 2.11 Calcium imaging

For calcium transient imaging in ventricular cardiac organoids, we primarily used the cell membrane permeable calcium ion fluorescent probe Fluo‐4, AM (Yeasen, 40704ES50). Specifically, 5 mM of Fluo‐4, AM storage solution was configured by adding DMSO, dispensed and stored at −20°C for a long time. It was diluted to 5 μM of Fluo‐4, AM working solution using HBSS solution prior to each experiment. Subsequent manipulations were performed directly in the ultra‐low adsorption 96‐well plates of culturing cardiac organoids. The original culture medium was first removed and washed three times using HBSS solution. After washing, 100 μL of Fluo‐4, AM working solution was added to each well and incubated for 30 min in 37°C. At the end of the process, it was washed three times using HBSS solution to remove the residual Fluo‐4 working solution. Next, 100 μL of HBSS solution was added and incubated for 20 min in a 37°C incubator to ensure complete de‐esterification of the AM moiety. Calcium transient images were captured using a Leica Incubator i8 live‐cell workstation at an excitation wavelength of 494 nm and an emission wavelength of 516 nm in a speed of 30 Hz, and the calcium signal changes were analysed with Image J (Version 1.54 g). Data was showed as F/F0 (F0 refers to baseline intensity, calculated by Image J) and plotted with Origin (Version 9.8.0.200).

### 2.12 RNA‐seq and analysis

Total RNA was extracted using Trizol reagent kit (Invitrogen) according to the manufacturer's protocol. RNA quality was assessed on an Agilent 2100 Bioanalyzer (Agilent Technologies) and checked using RNase free agarose gel electrophoresis. After total RNA was extracted, eukaryotic mRNA was enriched by Oligo (dT) beads. Then, the enriched mRNA was fragmented into short fragments using fragmentation buffer and reversely transcribed into cDNA by using NEBNext Ultra RNA Library Prep Kit for Illumina (New England Biolabs). The purified double‐stranded cDNA fragments were end repaired, A base added, and ligated to Illumina sequencing adapters. The ligation reaction was purified with the AMPure XP Beads (1.0X) and amplified by PCR. The resulting cDNA library was sequenced using Illumina Novaseq6000 by Gene Denovo Biotechnology Co (Guangzhou, China).

Principal component analysis (PCA) was performed with R package gmodels (http://www.r-project.org/) in this experience. RNAs differential expression analysis was performed by DESeq2^21^ software between two different groups. The genes/transcripts with the parameter of false discovery rate (FDR) below 0.05 and absolute fold change≥1.5 were considered differentially expressed genes (DEGs)/transcripts. Gene Ontology (GO) enrichment analysis provides all GO terms that significantly enriched in DEGs comparing to the genome background, and filter the DEGs that correspond to biological functions.^22^ Pathway enrichment analysis identified significantly enriched metabolic pathways or signal transduction pathways in DEGs comparing with the whole genome background in Kyoto Encyclopedia of Genes and Genomes (KEGG) database.^23^ STRING database (https://string-db.org/) was adopted to describe the signalling network among selected proteins. And the results were visualized with Cytoscape (Version 3.7.1; San Diego, CA, USA).

### 2.13 Statistical analysis

All results were presented as mean ± standard deviation. An unpaired *t*‐test was employed for comparing two groups. Statistical differences among the three groups were assessed using one‐way ANOVA, and the *p*‐values were adjusted using the Bonferroni test for multiple comparisons. For cell supernatant/protein/RNA analysis, each sample was extracted from 8 to 16 individual ventricular cardiac organoids in the same batch and *n* represents independent replicates. Data analysis and visualization were performed using GraphPad Prism software (Version 9.4.1; San Diego, CA, USA). A *p*‐value <0.05 was considered statistically significant, denoted by one asterisk (*) for *p* < 0.05, two asterisks (**) for *p* < 0.01 and three asterisks (***) for *p* < 0.001.

## 3 RESULTS

### 3.1 Generation of ventricular cardiac organoids from hPSCs by inhibiting RA signalling pathway

**FIGURE 1:**
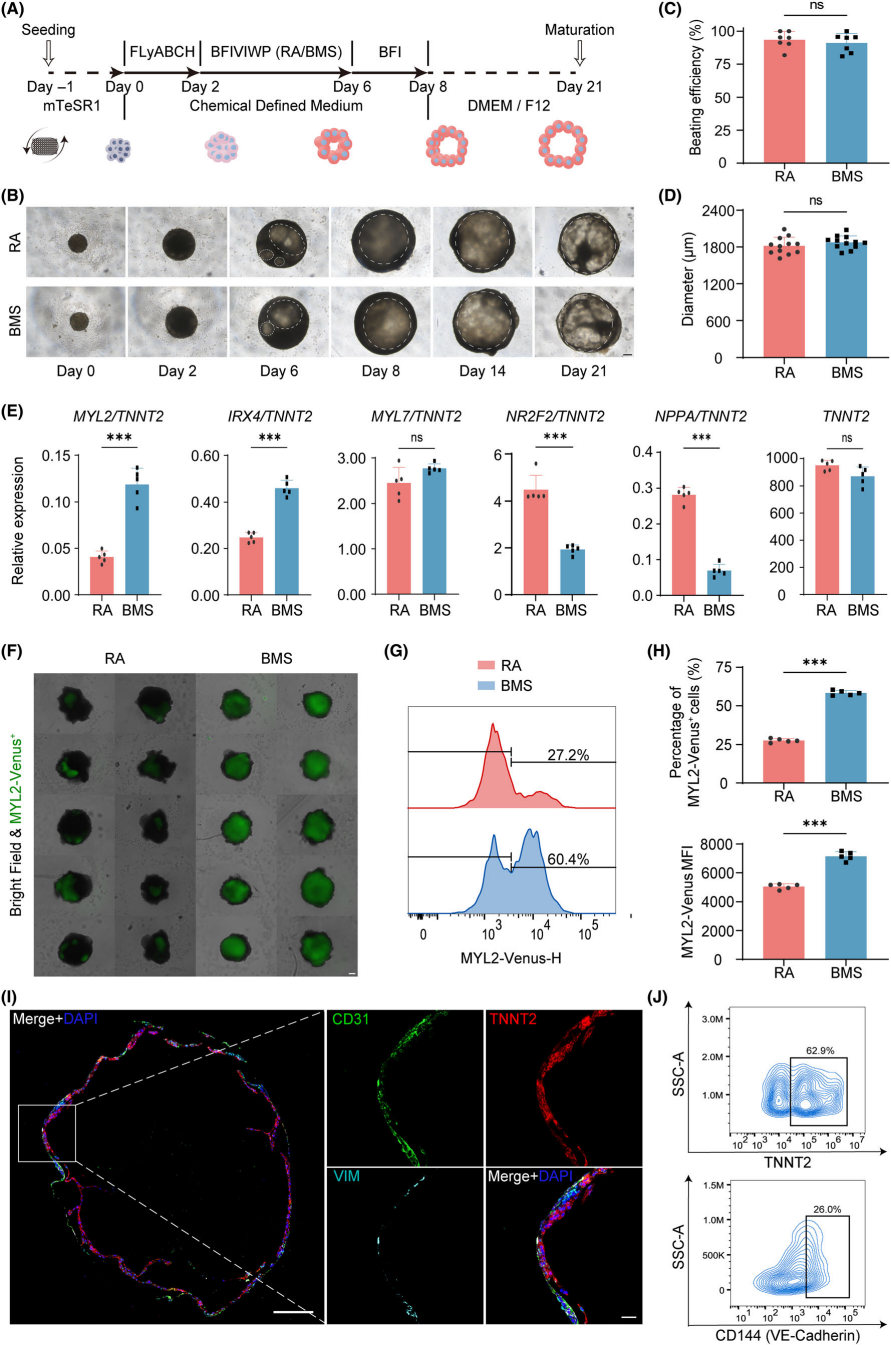
Derivation of human ventricular cardiac organoids. (A) Construction process of cardiac organoids with activated/inhibited retinoic acid (RA) signalling pathway. (B) Bright‐field microscopy images of RA and BMS treated cardiac organoids at different time points. The range of dotted boxes shows well‐organized chamber‐like structure. Scale bar, 200 μm. (C) Beating efficiency of RA and BMS treated groups on Day 21, *n* = 7 independent replicates. The proportions for each batch were calculated from 8 organoids. (D) Diameter of RA and BMS treated groups on Day 21, *n* = 12 independent replicates. (E) mRNA expression levels of RA and BMS treated groups on Day 8, ratios calculated from the expression values of *MYL2*, *IRX4*, *MYL7*, *NR2F2* and *NPPA* divided by the mean expression value of *TNNT2*, *n* = 5 independent replicates. (F) Fluorescence microscopy images of *MYL2‐Venus* hESCs reporter line‐constructed RA and BMS treated groups on Day 21. Scale bar, 200 μm. (G) Histogram of flow cytometry analysis for *MYL2‐Venus* hESCs reporter line‐constructed RA and BMS treated groups on Day 21. (H) Proportion of MYL2‐Venus‐positive cell populations and mean fluorescence intensity in flow cytometry analysis, *n* = 5 independent replicates. (I) Cell type distribution of chambered ventricular cardiac organoids obtained by inhibiting RA signalling pathway on Day 21. Scale bar (left), 200 μm. Scale bar (right), 40 μm. (J) Proportion of TNNT2 and CD144 positive cells for ventricular cardiac organoids by flow cytometry analysis. RA indicates retinoic acid, BMS indicates BMS493 and VIM indicates Vimentin. Bar and dot plot graphs show mean ± SD. Statistical significance was assessed by unpaired *t*‐test (***p < 0.001, ns indicates not significant).

Ventricular CMs were much more vulnerable than atrial CMs when I/R injury occurred. RA signalling has been extensively reported for fate determination of atrial CMs, which may bring bias for ischemic cardiomyopathy modelling by using atrial hPSCs‐CMs.^24^ Former reported chambered cardiac organoids applying RA were primarily composed of early MYL7^+^ CMs rather than ventricular‐specific MYL2^+^ CMs, limiting its potential application.^13^ Our repeated experiments also confirmed that on Day 8, MYL7^+^ CMs were observed in the RA‐induced chambered cardiac organoids (Video S1), while no MYL2^+^ CMs were detected (Figure S1A,D), indicating an early fate characteristic of CMs in former reported cardiac organoids. To promote ventricular specialization, we initially attempted to remove RA. It turned out that chambered cardiac organoids, primarily composed of CMs and ECs, still formed (Figure S1B,C), indicating RA signalling was not indispensable for cardiac organoid differentiation. To further detect and eliminate the effects of RA signalling, we developed two distinct protocols to activate or inhibit RA signalling pathway using RA or BMS493 (Figure 1A). No obvious differences in terms of the developmental status of cardiac organoids at various time points were observed between these two protocols (Figure 1B). Statistical analysis revealed no significant differences in beating efficiency and diameter of organoids on Day 21, as both groups exhibit over 90% beating organoids (Figure 1C) with diameter averaging about 1800 μm (Figure 1D). However, gene expression on Day 8, a stage when cardiac organoids had just started beating, revealed that BMS493 treated group exhibited higher expression of ventricular‐specific markers, such as *MYL2* and *IRX4*, compared to RA treated group. Atrial‐specific markers, such as *NR2F2* and *NPPA*, were higher in RA treated group, while differences in non‐specific markers *MYL7* and *TNNT2* were not observed (Figure 1E). Besides, IF staining proved higher MYL2 expression in BMS493 treated group (Figure S1D), indicating CMs undergoing a transition toward ventricular fate. For further confirmation, we conducted the same protocol on *MYL2‐Venus* hESCs reporter line, which expresses Venus only in MYL2^+^ ventricular‐specific CMs upon cardiac differentiation^18^ (Figure S1A). On Day 21, it became visible under the fluorescence microscope that MYL2 exhibit higher expression in BMS493 treated group (Figure 1F). Flow cytometry analysis further showed significant increases in MYL2‐Venus^+^ population and mean fluorescence intensity in BMS493 treated group compared to RA treated group (Figure 1G,H). IF staining revealed that with the BMS493 treatment, the CD31^+^ endothelial layer and TNNT2^+^ myocardial layer were clearly distinguishable, with a few number of fibroblasts expressing Vimentin (VIM), exhibiting a beating 3D structure of chambered cardiac organoids (Figure 1I, Video S2). Flow cytometry analysis showed that TNNT2^+^ CMs made up for approximately 62.9% of all cells, and CD144^+^ ECs for around 26.0% (Figure 1J) in these organoids. Thus, we have successfully established a differentiation protocol for the generation of chambered cardiac organoids with ventricular‐specific properties by inhibiting RA signalling pathway. For convenience, we named these organoids as ventricular cardiac organoids hereafter.

### 3.2 H/R simulates I/R injury in human ventricular cardiac organoids

**FIGURE 2:**
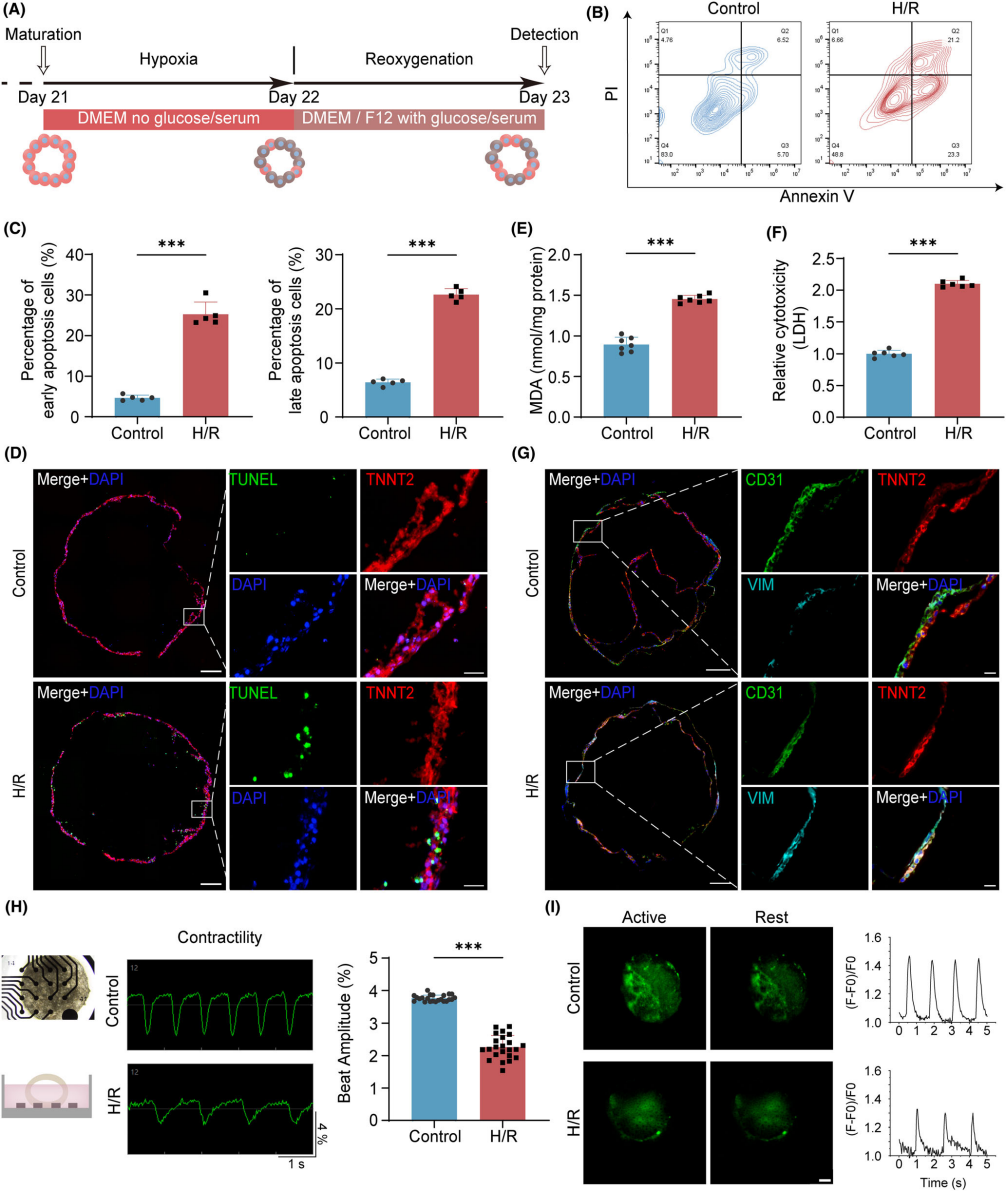
Hypoxia/reoxygenation (H/R)‐induced injury in human ventricular cardiac organoids. (A) Modelling process of H/R in ventricular cardiac organoids. (B) Contour plots of flow cytometry analysis for apoptotic cells in control and H/R groups by Annexin V‐FITC/PI‐PE staining. (C) Proportions of early and late apoptosis cells in flow cytometry analysis for control and H/R groups, *n* = 5 independent replicates. (D) Distribution of TNNT2 (red), TUNEL (green) and DAPI (blue) in control and H/R groups. Scale bars (left), 200 μm. Scale bars (right), 20 μm. (E) MDA concentration in control and H/R groups, *n* = 7 independent replicates. (F) Relative cytotoxicity of lactate dehydrogenase (LDH) in control and H/R groups, *n* = 6 independent replicates. (G) Distribution of CMs (TNNT2), ECs (CD31), and fibroblasts (VIM) in control and H/R groups. Scale bars (left), 200 μm. Scale bars (right), 20 μm. (H) Bright‐field microscopy images and schematic representation of MEA‐measured contractility and Beat Amplitude values (*n* = 23 electrodes per group) in control and H/R groups. (I) Representative calcium images at active and rest stages in control and H/R groups and the synchronized contraction was shown by calcium flux frequency. Results were expressed as F/F0 (F0 refers to baseline intensity). Scale bar, 200 μm. H/R indicates hypoxia/reoxygenation and VIM indicates Vimentin. Bar and dot plot graphs show mean ± SD. Statistical significance was assessed by unpaired *t*‐test (***p < 0.001).

In monolayer culture system, H/R modelling has been widely used,^25^ but usually, only CMs are involved, and phenotypes are therefore limited. Here, we performed H/R on our ventricular cardiac organoids, the faithful in vitro model to the native heart, to mimic I/R injury. To determine the optimal inducing time, we tried different durations for hypoxia and reoxygenation respectively (Figure S2A). It displayed that 24‐h hypoxia followed by 24‐h reoxygenation (Figure 2A) could coordinate the proportions of surviving cells, early apoptosis cells, and late apoptosis cells at approximately 50:25:22 (Figure 2B,C), ensuring adequate but not excessive damage, consistent with the characteristics of I/R injury (Video S3). To determine the primary cell types undergoing apoptosis, we co‐stained TUNEL with cell markers specific to CMs, ECs, or fibroblasts and quantified the proportion of apoptotic cells for each cell type (Figures 2D and S2B). The results indicate that CMs exhibit the highest proportion of apoptosis, followed by a lower proportion in ECs, while fibroblasts represent only a minimal fraction (Figure S2C,D). In addition, ventricular cardiac organoids undergoing injury released more MDA and LDH (Figure 2E,F), while expressing lower SOD (Figure S2E), indicating H/R injury in these organoids could give rise to oxidative stress. Moreover, IF staining of cardiac cell types showed that H/R injury disturbed the overall structure of ventricular cardiac organoids. CMs closer to ECs experienced some protection while the overall number of CMs decreased, besides, fluorescence intensity of fibroblasts‐specific VIM increased (Figures 2G and S2F). To further clarify whether fibroblasts were activated or not, we co‐stained fibroblast activation protein (FAP) and VIM in sections of ventricular cardiac organoids in control and H/R groups (Figure S2G). Results showed that there was significant co‐localization of the two proteins after H/R, suggesting the occurrence of fibrosis. In terms of electrophysiology, detection from MEA displayed the lower beat amplitude and spike amplitude with prolonged intervals (Figures 2H and S2H). Calcium imaging of myocardium in H/R group also had slower declines and decreased calcium influx frequency (Figures 2I and S2I). These findings indicate that ventricular cardiac organoids can response to H/R injury with increased cell apoptosis, elevated oxidative stress, disordered morphological structure and aberrated electrophysiological function, consistent with the pathophysiological activities of myocardial I/R injury.

### 3.3 RNA‐seq analysis reveals a potential role of IFN‐I in H/R‐induced ventricular cardiac organoids

**FIGURE 3:**
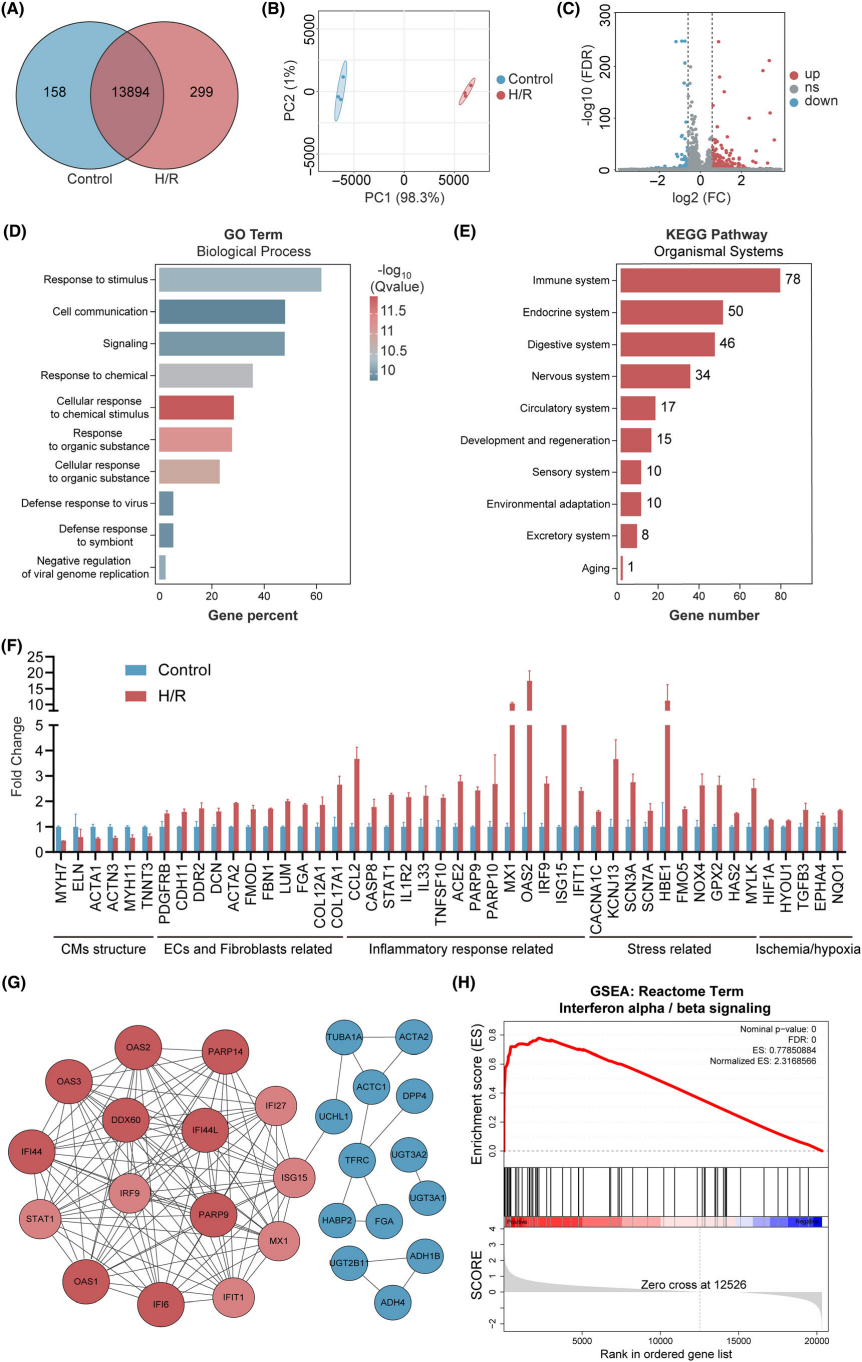
RNA‐seq analysis of human ventricular cardiac organoids in control and H/R groups. (A) All genes were identified in hypoxia/reoxygenation (H/R) and control groups by RNA‐seq. (B) PCA plot of gene expression for ventricular cardiac organoids in the control and H/R groups. (C) Volcano plot illustrating differentially expressed genes (DEGs) between the control and H/R groups (log_2_FC ≥1.5 and FDR<0.05). (D) Pathways in the Biological Process of GO enrichment analysis for upregulated DEGs in the H/R group. (E) Number of upregulated DEGs in the H/R group associated with pathways in various systems in KEGG enrichment analysis. (F) The fold change in DEGs related to CMs structural disruption, ECs stress, fibroblasts activation, response with inflammation and stress, and cardiac ischemia/hypoxia. (G) Protein–protein interaction network depicting the top 30 upregulated DEGs in the H/R group. (H) Gene Set Enrichment Analysis (GSEA) plot of the IFN‐I signalling pathway from the Reactome enrichment analysis. H/R indicates hypoxia/reoxygenation.

Afterwards, we performed RNA‐seq on ventricular cardiac organoids to further investigate the mechanism of H/R‐induced injury. A total of 13,894 genes were identified in control and H/R groups (Figure 3A). PCA demonstrated excellent batch‐to‐batch reproducibility, while showing significant inter‐group differences (Figure 3B). As depicted in the volcano plot and heatmap, a total of 527 DEGs have been identified, comprising 402 upregulated genes and 125 downregulated genes following H/R injury (Figures 3C and S3A). GO enrichment analysis was conducted on these DEGs, categorizing them based on biological processes, cellular components and molecular functions (Figure S3B). We noted that DEGs were enriched in some biological processes category that may involve in H/R injury, including response to stimulus, cell communication, and immune‐related reactions (Figure 3D). Furthermore, KEGG enrichment analysis identified a total of 274 enriched pathways (Figure S3C), with 78 genes out of the 527 DEGs enriching in immune system‐related pathways, surpassing the second‐ranking endocrine system (Figure 3E). Alterations in genes related to CMs structural disruption, ECs stress, fibroblasts activation and response with stress and inflammation were also evident among DEGs. Besides, key genes that mark cardiac ischemia/hypoxia, such as HIF1α and other related genes, were also elevated (Figure 3F). And there was a consistent trend in the corresponding terms in GO enrichment analysis (Figure S3D), demonstrating a genetic‐level similarity to characteristics of myocardial I/R injury. Of particular note, among the top 30 significantly upregulated genes, 15 were associated with the secretion of IFN‐I (Figure 3G), which was also validated in IFN‐I signalling pathway from the Reactome enrichment analysis (Figure 3H). These results indicate that in circumstances of in vitro cardiac I/R model, IFN‐I may play an important role in the occurrence and development of H/R‐induced injury.

### 3.4 THP‐1 cells co‐cultured with H/R‐induced ventricular cardiac organoids show crosstalk interaction

**FIGURE 4:**
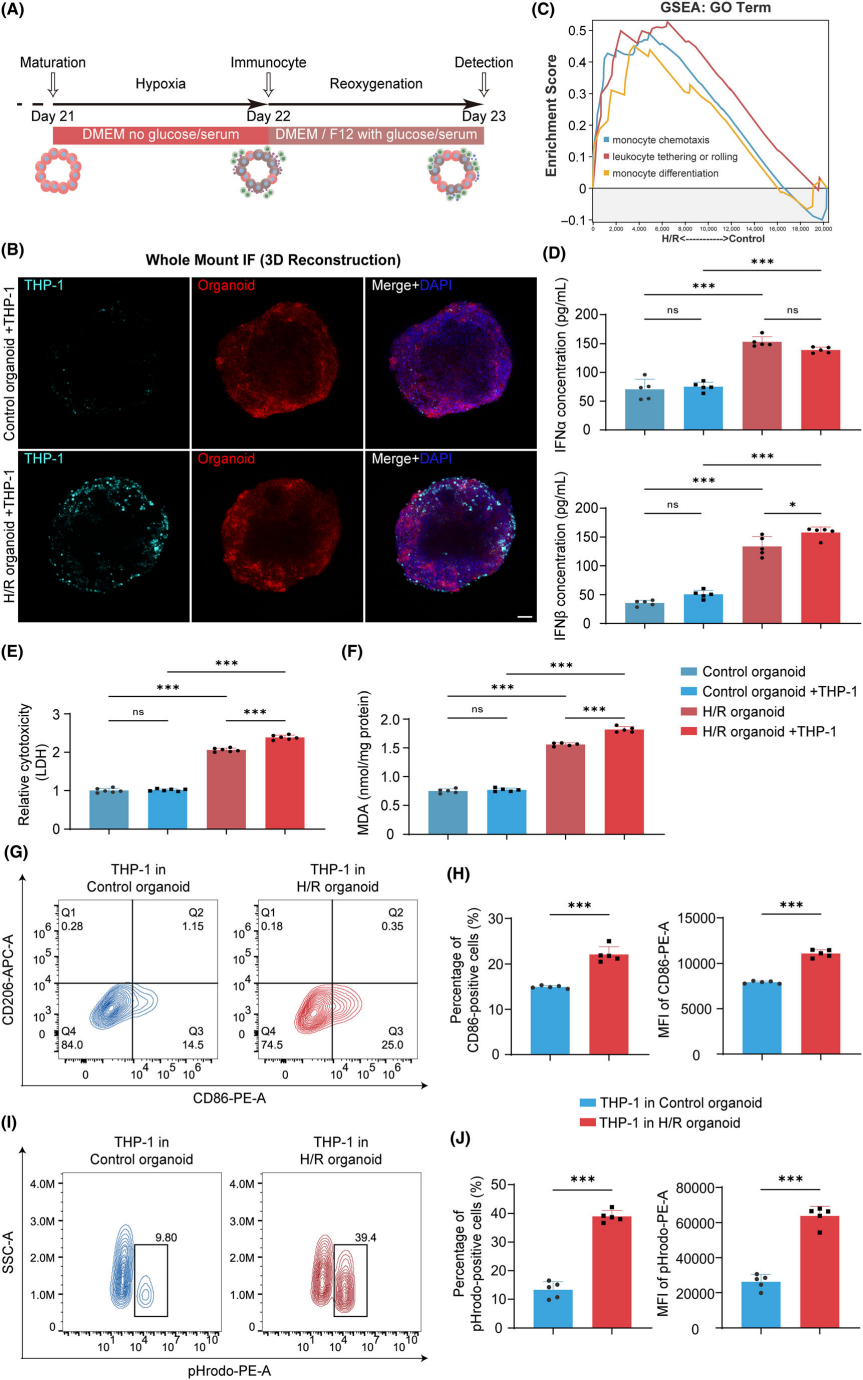
H/R induces inflammatory response between co‐cultured THP‐1 cells and ventricular cardiac organoids. (A) Co‐culture process of hypoxia/reoxygenation (H/R)‐induced ventricular cardiac organoids with THP‐1 cells. (B) Whole mount IF staining imaging of PKH26‐labelled THP‐1 cells and DIO‐labelled ventricular cardiac organoids after co‐culturing for 24 h. Scale bar, 100 μm. (C) Gene Set Enrichment Analysis plot of the monocyte chemotaxis and adhesion pathways from the GO enrichment analysis. (D) Secretion levels of IFN‐I subtypes α and β in control organoid, control organoid co‐culturing with THP‐1, H/R organoid and H/R organoid co‐culturing with THP‐1 groups, *n* = 5 independent replicates. (E) Relative cytotoxicity of LDH in control, control co‐culturing with THP‐1, H/R and H/R co‐culturing with THP‐1 groups, *n* = 6 independent replicates. (F) MDA concentration control, control co‐culturing with THP‐1, H/R and H/R co‐culturing with THP‐1 groups, *n* = 6 independent replicates. (G) Contour plots from flow cytometry analysis of THP‐1 cells co‐cultured with control and H/R‐induced ventricular cardiac organoids expressing M1 macrophage polarization marker CD86 and M2 macrophage polarization marker CD206. (H) Proportion and mean fluorescence intensity of CD86‐positive cell populations of THP‐1 cells co‐cultured with above two groups, *n* = 5. (I) Contour plots of flow cytometry analysis for THP‐1 cells co‐cultured with control and H/R‐induced ventricular cardiac organoids absorbing cytoplasmic acid dye pHrodo. (J) Proportion and mean fluorescence intensity of pHrodo‐positive cell populations, *n* = 5 independent replicates. H/R indicates hypoxia/reoxygenation. Bar and dot plot graphs show mean ± SD. Statistical significance was assessed by unpaired t‐test and one‐way ANOVA (*p < 0.05, ***p < 0.001).

In the context of H/R injury, elevated IFN‐I may be involved in the process of immune response. Except for the intrinsic changes within the heart, the activation of the immune response in situ has been regarded as critical pathological alterations during myocardial I/R injury.^2^ Of note, monocyte/macrophage involves in regulating acute inflammation response during reperfusion.^26^ Thus, to identify the role of monocyte/macrophage in H/R‐induced ventricular cardiac organoids, we introduced human monocytic cell line THP‐1 into the model during the reoxygenation phase (Figure 4A). Interestingly, whole mount IF staining imaging showed that THP‐1 cells could infiltrate into H/R‐induced organoids (Figure 4B), which might result from chemokines and pro‐inflammatory cytokines secreted by H/R‐induced ventricular cardiac organoids. Therefore, we evaluated classical factors such as *IL6*, *TNFα*, *CCL2* and *CCL4*. Indeed, the expression of these factors were elevated in H/R‐induced ventricular cardiac organoids before co‐culture with THP‐1 cells (Figure S4A). Monocyte chemotaxis or adhesion related pathways were also significantly enriched in H/R group of previous sequencing data (Figure 4C). We verified the secretion of the two main subtypes of IFN‐I, in which IFNα did not change significantly with the addition of THP‐1, while IFNβ was further elevated (Figure 4D). Furthermore, we found LDH and MDA release significantly increased, indicating that immune cells might exacerbate the oxidative stress (Figure 4E,F). Besides, THP‐1 cells co‐cultured with H/R‐induced ventricular cardiac organoids presented increased expression of macrophage‐specific marker *CD68* and M1‐like macrophage markers *CD86* and *CD274* (Figure S4B). Flow cytometry analysis showed more CD86^+^ cells (Figure 4G) and higher mean fluorescence intensity of CD86 protein (Figure 4H) in H/R‐induced ventricular cardiac organoids, indicating that THP‐1 cells underwent a transition toward the M1 phenotype. In addition to polarization, we have used the cytoplasmic acid dye pHrodo to find out the proportion of THP‐1 cells that develop into cells with phagocytic function, and the trend was consistent with the above (Figure 4I,J). Together, our data indicate the potential crosstalk between H/R‐induced ventricular cardiac organoids and monocytes/macrophages, which represents the activated inflammatory response, further confirming the similarity of H/R‐induced in vitro model to myocardial I/R injury.

### 3.5 Anifrolumab decreases IFN‐I secretion and related gene expression via blocking IFNAR1

**FIGURE 5:**
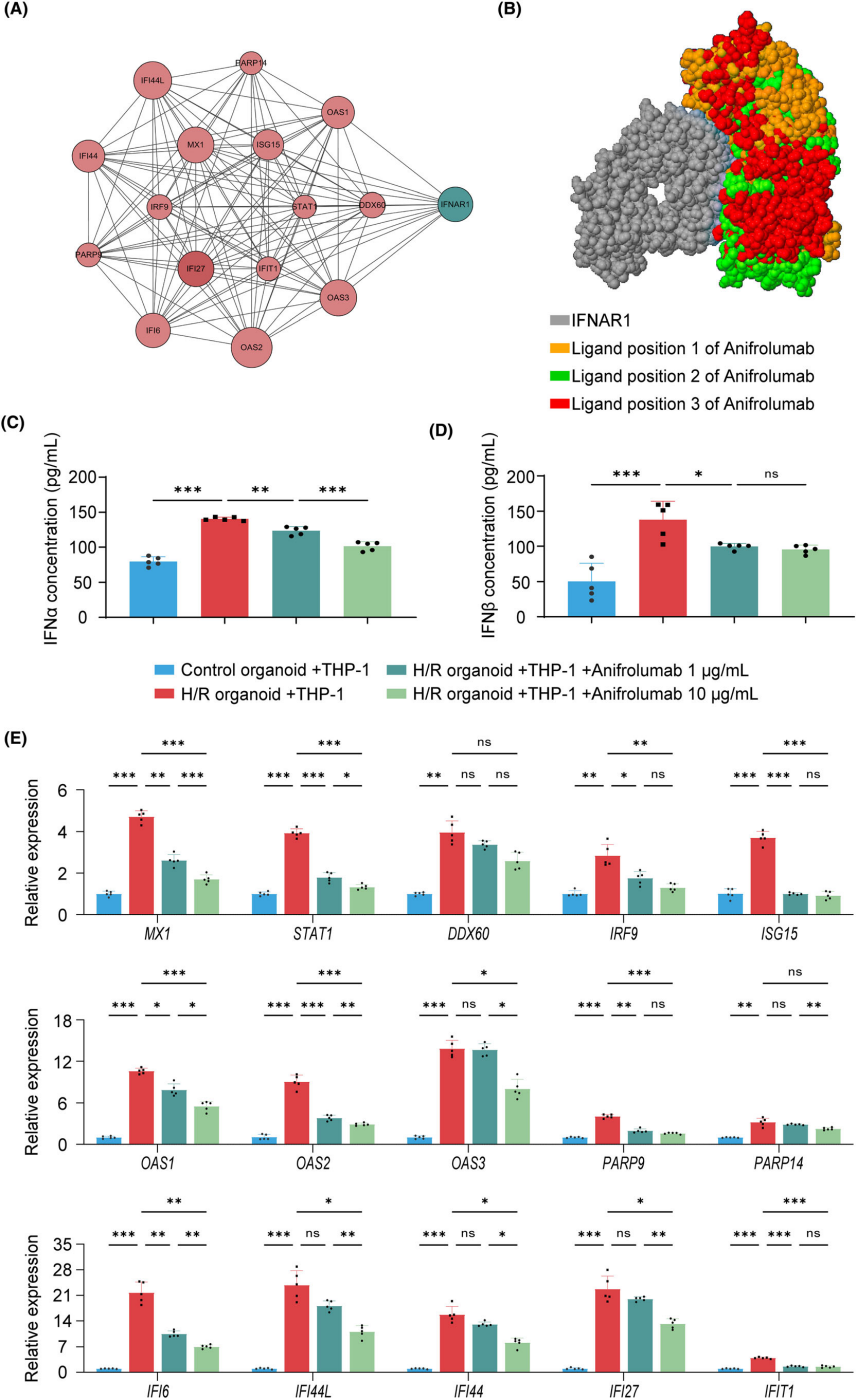
Anifrolumab suppresses the secretion of IFN‐I and the expression of related genes. (A) Protein–protein interaction network revealing the relationship between IFN‐I related genes among the top 30 upregulated genes and the Anifrolumab target, IFNAR1. (B) Ligand positions between IFNAR1 and Anifrolumab. (C, D) Secretion of IFNα and β in the culture medium of control organoid co‐culturing with THP‐1, hypoxia/reoxygenation (H/R) organoid co‐culturing with THP‐1, H/R organoid co‐culturing with THP‐1 + 1 μg/mL Anifrolumab group, and H/R organoid co‐culturing with THP‐1 + 10 μg/mL Anifrolumab group, *n* = 5 independent replicates. (E) mRNA expression levels of key IFN‐I related genes in ventricular cardiac organoids among the above four groups, *n* = 5 independent replicates. H/R indicates hypoxia/reoxygenation. Bar and dot plot graphs show mean ± SD. Statistical significance was assessed by one‐way ANOVA (*p < 0.05, **p < 0.01, ***p < 0.001, ns indicates not significant).

To investigate the regulatory role of IFN‐I in mediating the crosstalk between H/R‐induced ventricular cardiac organoids and THP‐1 cells, we used Anifrolumab, an FDA‐approved human monoclonal antibody that binds to the IFNα and β receptor subunit 1 (IFNAR1),^27^ to block the action of IFN‐I. IFNAR1 can activate multiple IFN‐I‐induced gene modules/pathways and form a self‐reinforcing positive feedback loop with IFN‐I‐secreting.^28^, ^29^ Protein–protein interaction network analysis revealed a relationship between Anifrolumab‐targeted IFNAR1 and IFN‐I related 15 genes among the top 30 upregulated genes of H/R‐induced ventricular cardiac organoids, and IFNAR1 was found to be directly associated with 12 of the 15 genes (Figure 5A). Protein docking analysis suggested that Anifrolumab effectively blocks IFNAR1 through multiple ligand positions (Figure 5B). Therefore, Anifrolumab may block the action of IFN‐I in H/R‐induced ventricular cardiac organoids by acting on IFNAR1.

In the co‐culture system of ventricular cardiac organoids with THP‐1 cells, we added Anifrolumab, using the concentration of 1 and 10 μg/mL during the reoxygenation phase as reported.^30^ Anifrolumab treatment efficiently reversed H/R‐induced increasing secretion of IFN‐I in the culture medium, including IFNα and β (Figure 5C,D), which may be due to their positive feedback loop. Moreover, the expression levels of most IFN‐I related genes among the top 30 upregulated genes, were downregulated following the Anifrolumab treatment (Figure 5E). Specifically, the expression level of *STAT1*, *IRF9* and *MX1*, which transduce the IFN‐I signal; *OAS1*, *OAS2*, *OAS3*, P*ARP9* and *PARP14*, which degrade nucleic acid following IFN‐I effects; *ISG15*, *IFI6*, *IFI44*, *IFI44L* and *IFIT1*, which regulate IFN‐I secretion, were markedly enhanced in the H/R group but they were significantly suppressed by Anifrolumab treatment. These results demonstrate that Anifrolumab decreases IFN‐I secretion and related gene expression via blocking IFNAR1. The drug responsiveness of H/R‐induced ventricular cardiac organoids and monocytes/macrophages co‐culture system revealed by Anifrolumab treatment also indicate broad application prospects of the above model for future anti‐inflammatory drug screening and target validation.

### 3.6 Anifrolumab attenuates H/R‐induced inflammation and oxidative stress in ventricular cardiac organoids and co‐cultured THP‐1 cells

**FIGURE 6:**
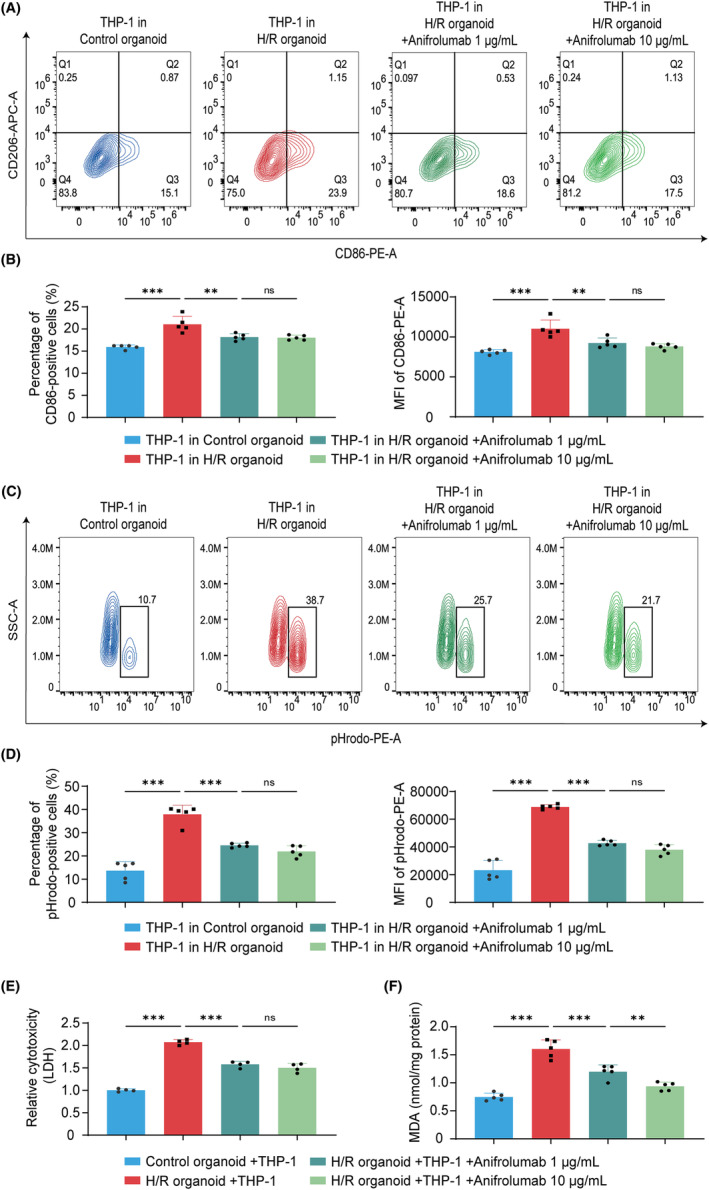
Anifrolumab intervenes hypoxia/reoxygenation (H/R)‐induced ventricular cardiac organoids and co‐cultured THP‐1 cells. (A) Contour plots from flow cytometry analysis of M1 macrophage polarization marker CD86 and M2 macrophage polarization marker CD206 in THP‐1 cells co‐cultured with control organoid, H/R organoid, H/R organoid +1 μg/mL Anifrolumab group, and H/R organoid +10 μg/mL Anifrolumab group. (B) Proportion and mean fluorescence intensity of CD86‐positive cell populations of THP‐1 cells co‐cultured with above four groups, *n* = 5. (C) Contour plots of flow cytometry analysis of absorbing cytoplasmic acid dye pHrodo for THP‐1 cells co‐cultured with above four groups. (D) Proportion and mean fluorescence intensity of pHrodo‐positive cell populations, *n* = 5 independent replicates. (E) Relative cytotoxicity of LDH in the control organoid co‐culturing with THP‐1, H/R organoid co‐culturing with THP‐1, H/R organoid co‐culturing with THP‐1 + 1 μg/mL Anifrolumab group, and H/R organoid co‐culturing with THP‐1 + 10 μg/mL Anifrolumab group, *n* = 4 independent replicates. (F) MDA concentration of ventricular cardiac organoids in the above four groups, *n* = 5. H/R indicates hypoxia/reoxygenation. Bar and dot plot graphs show mean ± SD. Statistical significance was assessed by one‐way ANOVA (**p < 0.01, ***p < 0.001, ns indicates not significant).

After confirming that the addition of Anifrolumab inhibited the expression of IFN‐I related genes, we then examined the corresponding changes in ventricular cardiac organoids and THP‐1 cells under the co‐culture system. Flow cytometry analysis showed that the proportion of CD86^+^ cells and the mean fluorescence intensity of CD86 protein, which had originally increased in THP‐1 cells after co‐culture with H/R‐induced ventricular cardiac organoids, were largely attenuated by Anifrolumab treatment (Figure 6A,B). Although there were still no CD206^+^ cells, the results indicate that the polarization of THP‐1 cells toward M1 phenotype macrophages is significantly inhibited. For pHrodo^+^ phagocytic monocytes, there was also a significant reduction in their proportion and mean fluorescence intensity after Anifrolumab treatment (Figure 6C,D). All these results suggest that the Anifrolumab treatment improves the pro‐inflammatory microenvironment and attenuates the acute inflammation response.

In addition, our previous data showed that the secretion levels of LDH and MDA were significantly higher after co‐culture with THP‐1 cells. However, after the addition of Anifrolumab, the pro‐inflammatory effect of THP‐1 cells was attenuated. We thus hypothesized that the level of oxidative stress would also be altered in the co‐culture system. So, we further examined the expression of LDH and MDA after the addition of Anifrolumab and the results showed that they both had different degrees of reduction (Figure 6E,F). Overall, these data suggest that Anifrolumab is an effective protein drug in attenuating the inflammatory response and oxidative stress damage for H/R‐induced ventricular cardiac organoids and THP‐1 cells. As Anifrolumab has already been approved by FDA and entered clinical trials, the newly discovered attenuation effect of Anifrolumab on H/R‐induced inflammation and oxidative stress may pave the way for future application in ischemic heart disease treatment.

## 4 DISCUSSION

In the differentiation protocols for monolayer CMs, many studies have revealed that the activation of RA signalling pathway during cardiac mesodermal stage would drive cells toward atrial‐like CMs.^24^ In contrast, the use of BMS493 to inhibit RA signalling pathway could take cells differentiate more toward ventricular‐like subtypes.^31^ Recently, by activating RA signalling pathway or not, atrial or ventricular heart organoids with cavities and hole structures were reported, but the so‐called chamber formation was not satisfactory in terms of cavity size and integrity.^32^ In 2021, Hofbauer et al. successfully developed the first self‐organized and chambered cardiac organoid, which shows early left ventricular chamber‐like characteristics like MYL7 expression.^13^ Latest research also reported multi‐chambered cardiac organoids, where ventricular‐CMs were obtained by fostering in a maturative culture environment.^33^ In this study, we first confirmed that RA is not indispensable for cardiac organoid formation. Furthermore, to reveal the role of RA signalling pathway more clearly, we set up two groups with adding RA or BMS493, to activate or inhibit it respectively. And separate vehicle group was not set up, because this is insufficient to conclusively determine whether downstream RA signalling pathways are affected. From the results, by inhibiting RA signalling with BMS493 during cardiac mesodermal stage, we established a stable protocol to derive human ventricular cardiac organoids with well‐organized chamber‐like structure enveloped by CMs, ECs and fibroblasts, indicating early inhibition of RA signalling is more streamlined and rapid for ventricular cardiac organoid construction.

Solid cardiac organoids were reported to model myocardial infarction in an oxygen‐diffusion gradient as the innermost cells receive the lowest oxygen supply.^16^ By contrast, chamber formation provides the equal stimulus to both the inner and outer layer cells of ventricular cardiac organoids, resulting in improved homogeneity as a whole and making it more reliable for mimicking ischemic heart diseases. Another study used CoCl_2_ as an inducer of hypoxia for modelling,^34^ while we believe that direct control of oxygen is more suitable for pathological conditions. In order to best simulate myocardial I/R injury, we refer to several modelling methods of monolayer CMs,^35^, ^36^ combined with hypoxia modelling protocol using 3D organoids,^37^ and finally determined H/R stimulation as the modelling method. It eventually resulted significant apoptosis of CMs. For pathological remodelling process, although fibroblasts were not the major non‐myocardial component, we found activation of fibrosis was evident after H/R injury. In addition, our model reflected the impact of H/R‐induced injury on cardiac electrophysiology functions, including calcium influx frequency decrease, beating spike amplitude reduction and contractility decline. These data collectively validated the resemblance of H/R‐induced injury in ventricular cardiac organoids to substitute myocardial I/R injury in preclinical study.

Correspondingly, according to our RNA‐seq data, a large number of DEGs were significantly enriched in cellular stress and immune‐related pathways. Though due to the challenges in obtaining primary human samples, the trends reflected in the CMs structural disruption, ECs stress, fibroblasts activation, and response with stress and inflammation, were consistent with in vivo pathological changes.^38^ Furthermore, we have noticed that myocardial I/R injury in vivo not only elicit pathological alterations in situ but also involves the recruitment of immune cells from the peripheral blood.^39^ Clinical evidence demonstrated that a more rapid reduction in circulation monocytes after myocardial I/R injury is associated with a larger infarct size and poorer left ventricular function.^40^ So far, there have been no humanized model to mimic this process, which limits the mechanistic analysis of myocardial I/R injury progression. For this, we would like to introduce human monocytes into the ventricular cardiac organoid culture system. Considering that there is not yet a widely accepted hiPSCs‐monocytes differentiation protocol, we used the THP‐1 cells, which are widely recognized in the field of immunology as a cell line to simulate peripheral blood monocytes. The results visibly demonstrated an obvious adhesion of THP‐1 cells to the surface of injured ventricular cardiac organoids, which aligned perfectly with the in vivo phenomenon where immune cells undergo chemotaxis and adhesion to the vascular ECs.^41^ The co‐culture system with THP‐1 cells also amplified oxidative stress damage in the ventricular cardiac organoids themself, in the same way that inflammatory responses and heart injury act mutually in vivo. These findings unravelled the superiority of 3D ventricular cardiac organoids in disease simulation, as it can incorporate an external immune system component.

Of note, in transcriptome data, IFN‐I drew our attention as it was associated with some of the most significantly altered genes and corresponded to previous reports in animal cardiac injury.^42^, ^43^, ^44^, ^45^ A recent study also found that an intercellular self‐stimulating inflammatory circuit between cardiac fibroblasts and macrophages via IFN‐IFNAR‐p‐STAT1 inhibits cardiac reprogramming in vivo.^29^ ECs also have been reported to have the ability to secrete IFN‐I, although this has not been confirmed in myocardial I/R injury.^46^ Thus, we verified the potential of an antagonist of IFN‐I signaling, Anifrolumab, in alleviating inflammation damage and reducing recruitment of immune cells. Actually, there have been instance of using antibodies antagonizing IFN to prompt cardiac repair post heart injury.^42^ As a drug that has already entered clinical trials, Anifrolumab's safety has been confirmed in systemic lupus erythematosus.^47^ Therefore, we anticipate future comprehensive investigations into the in vivo dosage, pharmacokinetics, and administration methods of Anifrolumab for I/R injury in the heart. In addition, previous studies in mice have reported that in situ immune cells activated the secretion of IFN‐I after cardiac injury and exacerbate the damage.^35^ Considering the cell types within our ventricular cardiac organoids, we assumed that ECs and fibroblasts are responsible for this phenomenon. It has been reported that ECs and fibroblasts are capable of secreting IFN‐I, although this has not garnered enough attention in myocardial I/R injury.^29^, ^46^, ^48^

In summary, we have successfully developed ventricular cardiac organoids and established a H/R‐induced model to simulate myocardial I/R injury. Innovatively, we introduced immune cells to replicate the activation of inflammatory response. Ultimately, based on RNA‐seq, we identified a potential therapeutic drug, Anifrolumab, for the attenuation of immune cells mediated inflammatory response and reperfusion induced oxidative stress. In the current landscape where organ chips have received FDA approval for use in drug experiments,^49^ we believe that this ventricular cardiac organoid model can be utilized for large‐scale drug screening and become an integral component for preclinical researches. Unfortunately, due to technical limitations, there is room for improvement in characterizing biomechanics changes and detailed electrophysiological alterations, such as early after depolarization (EAD) or delayed after depolarization (DAD).^50^ Additionally, as there is no extra addition of matrix gels to the differentiation environment, which was reported to be important for tube‐formation or self‐assembled vascularization of ECs in cardiac microtissues,^19^ vascular regeneration after inflammatory infiltration was not evaluated in our ventricular cardiac organoids. It should be of great significance to further investigate the thoroughly vascularized cardiac organoids, which is expected to be independent of culture medium permeation and play a better role in drug screening or prognosis predicting on myocardial I/R injury or other pathological lesions.

## AUTHOR CONTRIBUTIONS

Y. L., Z. L. and H. Y. supervised and conceived the study; Y. L. and L. Z. were responsible for experiments designs; L. Z. carried out experiments and drafted the paper; Y. L. contributed to data interpretation and manuscript preparation; W. J. provided the ZB11AOF hiPSCs; H. Y. and P. Z. established the *MYL2‐Venus* hESCs cell line and participated partial experiments designs; Y. J. contributed to data interpretation; W. L. and J. L. provided technical support in data collection; Y. L., H. Y. and P. Z. revised the manuscript. All authors read and approved the final manuscript.

## CONFLICT OF INTEREST STATEMENT

The authors declare no conflicts of interest.

## Supporting information


Figure S1. Characteristics of hPSCs and their derived cardiac organoids.
(A) Bright‐field images of ZB11AOF hiPSC and *MYL2‐Venus* hESC. Scale bars, 100 μm. (B) Bright‐field microscopy images of +RA and ‐RA treated cardiac organoids at Day 6. Scale bar, 200 μm. (C) Immunofluorescence staining of TNNT2 (red) and CD31 (green) for cardiac organoids fabricated in +RA and ‐RA treated groups on Day 8. Scale bars, 100 μm. (D) Immunofluorescence staining of MYL2 (red) and MYL7 (green) for cardiac organoids fabricated in RA and BMS treated groups on Day 8. Scale bars, 100 μm. +RA and RA indicate adding retinoic acid, −RA indicates removing retinoic acid, BMS indicates BMS493.

Figure S2. Determination of H/R‐induced injury in ventricular cardiac organoids.
(A) Proportions of early and late apoptotic cells for different time ratio of hypoxia and reoxygenation by flow cytometry analysis. (B) Distribution of CD31 (red), VIM (cyan), TUNEL (green) and DAPI (blue) in control and H/R groups. Scale bars (left), 200 μm. Scale bars (right), 20 μm. (C) Percentage of TUNEL‐positive cells, *n* = 4 independent replicates. (D) Percentage of different cell types among TUNEL‐positive cells, *n* = 4 independent replicates. (E) SOD activity in control and H/R groups, *n* = 8 independent replicates per group. (F) Flurorescence intensity of TNNT2 and VIM in Figure 2G, *n* = 3 images per group. (G) Distribution of FAP (red), VIM (green) and DAPI (blue) in control and H/R groups. Scale bars (left), 200 μm. Scale bars (right), 20 μm. (H) MEA‐measured field potential and spike amplitude values (*n* = 36 electrodes) in control and H/R groups. (I) Interval time (s) between every beating, *n* = 16 organoids. H/R indicates hypoxia/reoxygenation and VIM indicates Vimentin. Bar and dot plot graphs show mean ± SD. Statistical significance was assessed by unpaired *t*‐test (**p < 0.01 and ***p < 0.001).

Figure S3. Heatmap and GO/KEGG analysis for DEGs.
(A) Heatmap displaying the expression patterns of differentially expressed genes. (B) GO enrichment analysis based on DEGs between Control and H/R group. (C) KEGG enrichment analysis based on DEGs between Control and H/R group. (D) GSEA plot of pathways related to CMs structural disruption, ECs stress, fibroblasts activation, and response with stress and inflammation from the GO enrichment analysis. H/R indicates hypoxia/reoxygenation.

Figure S4. Expression changes of inflammatory‐related genes in ventricular cardiac organoids and co‐cultured THP‐1 cells.
(A) mRNA expression levels of pro‐inflammatory factors in control and H/R groups of ventricular cardiac organoids, *n* = 5. (B) mRNA expression levels of M1 macrophage polarization markers in THP‐1 cells co‐cultured with control and H/R‐induced ventricular cardiac organoids, *n* = 5. H/R indicates hypoxia/reoxygenation. Bar and dot plot graphs show mean ± SD. Statistical significance was assessed by unpaired *t*‐test and one‐way ANOVA (**p < 0.01, ***p < 0.001, ns indicates not significant).

**Video S1.** Human chambered cardiac organoids induced by RA show spontaneous beating, related to Figure 1.

**Video S2.** Human ventricular cardiac organoids induced by BMS493 show spontaneous beating, related to Figure 1.

**Video S3.** H/R‐induced human ventricular cardiac organoids show weakened beating property, related to Figure 2.

**Table S1.** Primer sets used for qPCR experiments.

## Data Availability

RNA sequencing data generated and used by this study are deposited at NCBI Sequence Read Archive (SRA). Datasets for RNA sequencing of ventricular cardiac organoids are available at NCBI‐SRA with the accession number PRJNA1068258. Other data that support the findings of this study are available from the corresponding authors upon reasonable request.

## References

[1] Reed GW , Rossi JE , Cannon CP . Acute myocardial infarction. Lancet. 2017;389:197‐210. doi:10.1016/s0140-6736(16)30677-8 27502078

[2] Heusch G , Gersh BJ . The pathophysiology of acute myocardial infarction and strategies of protection beyond reperfusion: a continual challenge. Eur Heart J. 2017;38:774‐784. doi:10.1093/eurheartj/ehw224 27354052

[3] Fröhlich GM , Meier P , White SK , Yellon DM , Hausenloy DJ . Myocardial reperfusion injury: looking beyond primary PCI. Eur Heart J. 2013;34:1714‐1722. doi:10.1093/eurheartj/eht090 23536610

[4] Cung TT , Morel O , Cayla G , et al. Cyclosporine before PCI in patients with acute myocardial infarction. N Engl J Med. 2015;373:1021‐1031. doi:10.1056/NEJMoa1505489 26321103

[5] Hahn JY , Song YB , Kim EK , et al. Ischemic postconditioning during primary percutaneous coronary intervention: the effects of postconditioning on myocardial reperfusion in patients with ST‐segment elevation myocardial infarction (POST) randomized trial. Circulation. 2013;128:1889‐1896. doi:10.1161/circulationaha.113.001690 24068776

[6] Engstrøm T , Kelbæk H , Helqvist S , et al. Effect of ischemic postconditioning during primary percutaneous coronary intervention for patients with ST‐segment elevation myocardial infarction: a randomized clinical trial. JAMA Cardiol. 2017;2:490‐497. doi:10.1001/jamacardio.2017.0022 28249094 PMC5814983

[7] Hay M , Thomas DW , Craighead JL , Economides C , Rosenthal J . Clinical development success rates for investigational drugs. Nat Biotechnol. 2014;32:40‐51. doi:10.1038/nbt.2786 24406927

[8] Odom DT , Dowell RD , Jacobsen ES , et al. Tissue‐specific transcriptional regulation has diverged significantly between human and mouse. Nat Genet. 2007;39:730‐732. doi:10.1038/ng2047 17529977 PMC3797512

[9] Haghighi K , Kolokathis F , Pater L , et al. Human phospholamban null results in lethal dilated cardiomyopathy revealing a critical difference between mouse and human. J Clin Invest. 2003;111:869‐876. doi:10.1172/jci17892 12639993 PMC153772

[10] Rahman A , Li Y , Chan TK , et al. Large animal models of cardiac ischemia‐reperfusion injury: where are we now? Zool Res. 2023;44:591‐603. doi:10.24272/j.issn.2095-8137.2022.487 37147910 PMC10236300

[11] Lian X , Zhang J , Azarin SM , et al. Directed cardiomyocyte differentiation from human pluripotent stem cells by modulating Wnt/β‐catenin signaling under fully defined conditions. Nat Protoc. 2013;8:162‐175. doi:10.1038/nprot.2012.150 23257984 PMC3612968

[12] Wnorowski A , Yang H , Wu JC . Progress, obstacles, and limitations in the use of stem cells in organ‐on‐a‐chip models. Adv Drug Deliv Rev. 2019;140:3‐11. doi:10.1016/j.addr.2018.06.001 29885330 PMC6281815

[13] Hofbauer P , Jahnel SM , Papai N , et al. Cardioids reveal self‐organizing principles of human cardiogenesis. Cell. 2021;184:3299‐3317.e22. doi:10.1016/j.cell.2021.04.034 34019794

[14] Lewis‐Israeli YR , Wasserman AH , Gabalski MA , et al. Self‐assembling human heart organoids for the modeling of cardiac development and congenital heart disease. Nat Commun. 2021;12:5142. doi:10.1038/s41467-021-25329-5 34446706 PMC8390749

[15] Lee A , Hudson AR , Shiwarski DJ , et al. 3D bioprinting of collagen to rebuild components of the human heart. Science. 2019;365:482‐487. doi:10.1126/science.aav9051 31371612

[16] Richards DJLY , Kerr CM , Yao J , Beeson GC , Coyle RC , et al. Human cardiac organoids for the modelling of myocardial infarction and drug cardiotoxicity. Nat Biomed Eng. 2020;4:446‐462. doi:10.1038/s41551-020-0539-4 32284552 PMC7422941

[17] Hu W , Lazar MA . Modelling metabolic diseases and drug response using stem cells and organoids. Nat Rev Endocrinol. 2022;18:744‐759. doi:10.1038/s41574-022-00733-z 36071283 PMC9449917

[18] Luo XL , Zhang P , Liu X , et al. Myosin light chain 2 marks differentiating ventricular cardiomyocytes derived from human embryonic stem cells. Pflug Arch Eur J Phy. 2021;473:991‐1007. doi:10.1007/s00424-021-02578-3 34031754

[19] Liu Y , Zhang Y , Mei T , et al. hESCs‐Derived Early Vascular Cell Spheroids for Cardiac Tissue Vascular Engineering and Myocardial Infarction Treatment. *Advanced Science* . Adv Sci. 2022;9:e2104299. doi:10.1002/advs.202104299 PMC8948571 35092352

[20] Qin XY , Shen HH , Zhang XY , et al. Hypoxia‐mediated chemotaxis and residence of macrophage in decidua by secreting VEGFA and CCL2 during normal pregnancy. Reproduction. 2023;165:543‐555. doi:10.1530/rep-22-0473 36809184

[21] Love MI , Huber W , Anders S . Moderated estimation of fold change and dispersion for RNA‐seq data with DESeq2. Genome Biol. 2014;15:550. doi:10.1186/s13059-014-0550-8 25516281 PMC4302049

[22] Ashburner M , Ball CA , Blake JA , et al. Gene ontology: tool for the unification of biology. The Gene Ontology Consortium Nature Genetics. 2000;25:25‐29. doi:10.1038/75556 10802651 PMC3037419

[23] Kanehisa M , Goto S . KEGG: kyoto encyclopedia of genes and genomes. Nucleic Acids Res. 2000;28:27‐30. doi:10.1093/nar/28.1.27 10592173 PMC102409

[24] Lee JH , Protze SI , Laksman Z , Backx PH , Keller GM . Human pluripotent stem cell‐derived atrial and ventricular cardiomyocytes develop from distinct mesoderm populations. Cell Stem Cell. 2017;21:179‐194.e4. doi:10.1016/j.stem.2017.07.003 28777944

[25] Häkli M , Kreutzer J , Mäki AJ , et al. Human induced pluripotent stem cell‐based platform for modeling cardiac ischemia. Sci Rep. 2021;11:4153. doi:10.1038/s41598-021-83740-w 33603154 PMC7893031

[26] Marinković G , Grauen Larsen H , Yndigegn T , et al. Inhibition of pro‐inflammatory myeloid cell responses by short‐term S100A9 blockade improves cardiac function after myocardial infarction. Eur Heart J. 2019;40:2713‐2723. doi:10.1093/eurheartj/ehz461 31292614

[27] Mullard A . FDA approves AstraZeneca's anifrolumab for lupus. Nat Rev Drug Discov. 2021;20:658. doi:10.1038/d41573-021-00139-y 34363027

[28] Baker T , Sharifian H , Newcombe PJ , et al. Type I interferon blockade with anifrolumab in patients with systemic lupus erythematosus modulates key immunopathological pathways in a gene expression and proteomic analysis of two phase 3 trials. Ann Rheum Dis. 2024;83:1018‐1027. doi:10.1136/ard-2023-225445 38569851 PMC12056589

[29] Wang H , Yang J , Cai Y , Zhao Y . Macrophages suppress cardiac reprogramming of fibroblasts in vivo via IFN‐mediated intercellular self‐stimulating circuit. Protein Cell. 2024:pwae013. doi:10.1093/procel/pwae013 PMC11637486 38530808

[30] Riggs JM , Hanna RN , Rajan B , et al. Characterisation of anifrolumab, a fully human anti‐interferon receptor antagonist antibody for the treatment of systemic lupus erythematosus. Lupus Sci Med. 2018;5:e000261. doi:10.1136/lupus-2018-000261 29644082 PMC5890856

[31] Zhang Q , Jiang J , Han P , et al. Direct differentiation of atrial and ventricular myocytes from human embryonic stem cells by alternating retinoid signals. Cell Res. 2011;21:579‐587. doi:10.1038/cr.2010.163 21102549 PMC3203651

[32] Feng W , Schriever H , Jiang S , et al. Computational profiling of hiPSC‐derived heart organoids reveals chamber defects associated with NKX2‐5 deficiency. Commun Bio. 2022;5:399. doi:10.1038/s42003-022-03346-4 35488063 PMC9054831

[33] Schmidt C , Deyett A , Ilmer T , et al. Multi‐chamber cardioids unravel human heart development and cardiac defects. Cell. 2023;186:5587‐5605.e27. doi:10.1016/j.cell.2023.10.030 38029745

[34] Song M , Choi DB , Im JS , et al. Modeling acute myocardial infarction and cardiac fibrosis using human induced pluripotent stem cell‐derived multi‐cellular heart organoids. Cell Death Dis. 2024;15:308. doi:10.1038/s41419-024-06703-9 38693114 PMC11063052

[35] Hidalgo A , Glass N , Ovchinnikov D , et al. Modelling ischemia‐reperfusion injury (IRI) in vitro using metabolically matured induced pluripotent stem cell‐derived cardiomyocytes. APL Bioeng. 2018;2:026102. doi:10.1063/1.5000746 31069299 PMC6481709

[36] Ward MC , Gilad Y . A generally conserved response to hypoxia in iPSC‐derived cardiomyocytes from humans and chimpanzees. Elife. 2019;8:8. doi:10.7554/eLife.42374 PMC6538380 30958265

[37] Sharma P , Liu Chung Ming C , Wang X , et al. Biofabrication of advanced in vitro 3D models to study ischaemic and doxorubicin‐induced myocardial damage. Biofabrication. 2022;14:14. doi:10.1088/1758-5090/ac47d8 34983029

[38] Gunata M , Parlakpinar H . A review of myocardial ischaemia/reperfusion injury: pathophysiology, experimental models, biomarkers, genetics and pharmacological treatment. Cell Biochem Funct. 2021;39:190‐217. doi:10.1002/cbf.3587 32892450

[39] Ruparelia N , Godec J , Lee R , et al. Acute myocardial infarction activates distinct inflammation and proliferation pathways in circulating monocytes, prior to recruitment, and identified through conserved transcriptional responses in mice and humans. Eur Heart J. 2015;36:1923‐1934. doi:10.1093/eurheartj/ehv195 25982896 PMC4571177

[40] Marsh SA , Park C , Redgrave RE , et al. Rapid fall in circulating non‐classical monocytes in ST elevation myocardial infarction patients correlates with cardiac injury. FASEB J. 2021;35:e21604. doi:10.1096/fj.202100240R 33913566

[41] Han Y , Liao X , Gao Z , et al. Cardiac troponin I exacerbates myocardial ischaemia/reperfusion injury by inducing the adhesion of monocytes to vascular endothelial cells via a TLR4/NF‐κB‐dependent pathway. Clin Sci. 2016;130:2279‐2293. doi:10.1042/cs20160373 27682003

[42] King KR , Aguirre AD , Ye YX , et al. IRF3 and type I interferons fuel a fatal response to myocardial infarction. Nat Med. 2017;23:1481‐1487. doi:10.1038/nm.4428 29106401 PMC6477926

[43] Lai L , Zhang A , Yang B , Charles EJ , Kron IL , Yang Z . Plasmacytoid dendritic cells mediate myocardial ischemia/reperfusion injury by secreting type I interferons. J Am Heart Assoc. 2021;10:e020754. doi:10.1161/jaha.121.020754 34325534 PMC8475660

[44] Calcagno DM , Ng RP Jr , Toomu A , et al. The myeloid type I interferon response to myocardial infarction begins in bone marrow and is regulated by Nrf2‐activated macrophages. Sci Immun. 2020;5:5. doi:10.1126/sciimmunol.aaz1974 PMC7808338 32978242

[45] Luo XL , Jiang Y , Li Q , et al. hESC‐Derived Epicardial Cells Promote Repair of Infarcted Hearts in Mouse and Swine. Adv Sci. 2023;10:e2300470. doi:10.1002/advs.202300470 PMC10520683 37505480

[46] Parra‐Izquierdo I , Sánchez‐Bayuela T , López J , et al. Interferons are pro‐inflammatory cytokines in sheared‐stressed human aortic valve endothelial cells. Int J Mol Sci. 2021;22:22. doi:10.3390/ijms221910605 PMC8508640 34638942

[47] Miyazaki Y , Funada M , Nakayamada S , et al. Safety and efficacy of anifrolumab therapy in systemic lupus erythematosus in real‐world clinical practice: LOOPS registry. Rheumatology (Oxford). 2024;63(9):2345‐2354. doi:10.1093/rheumatology/kead568 37934129

[48] Bolivar S , Espitia‐Corredor JA , Olivares‐Silva F , et al. In cardiac fibroblasts, interferon‐beta attenuates differentiation, collagen synthesis, and TGF‐β1‐induced collagen gel contraction. Cytokine. 2021;138:155359. doi:10.1016/j.cyto.2020.155359 33160814

[49] Wadman M . FDA no longer has to require animal testing for new drugs. Science. 2023;379:127‐128. doi:10.1126/science.adg6276 36634170

[50] Lyu Q , Gong S , Lees JG , et al. A soft and ultrasensitive force sensing diaphragm for probing cardiac organoids instantaneously and wirelessly. Nat Commun. 2022;13:7259. doi:10.1038/s41467-022-34860-y 36433978 PMC9700778

