# Supplementary figures and images for: Modelling myocardial ischemia/reperfusion injury with inflammatory response in human ventricular cardiac organoids
Source: Cell Prolif. 2024 Oct 8;58(3):e13762. doi: 10.1111/cpr.13762 (PMC11882745; doi:10.1111/cpr.13762)

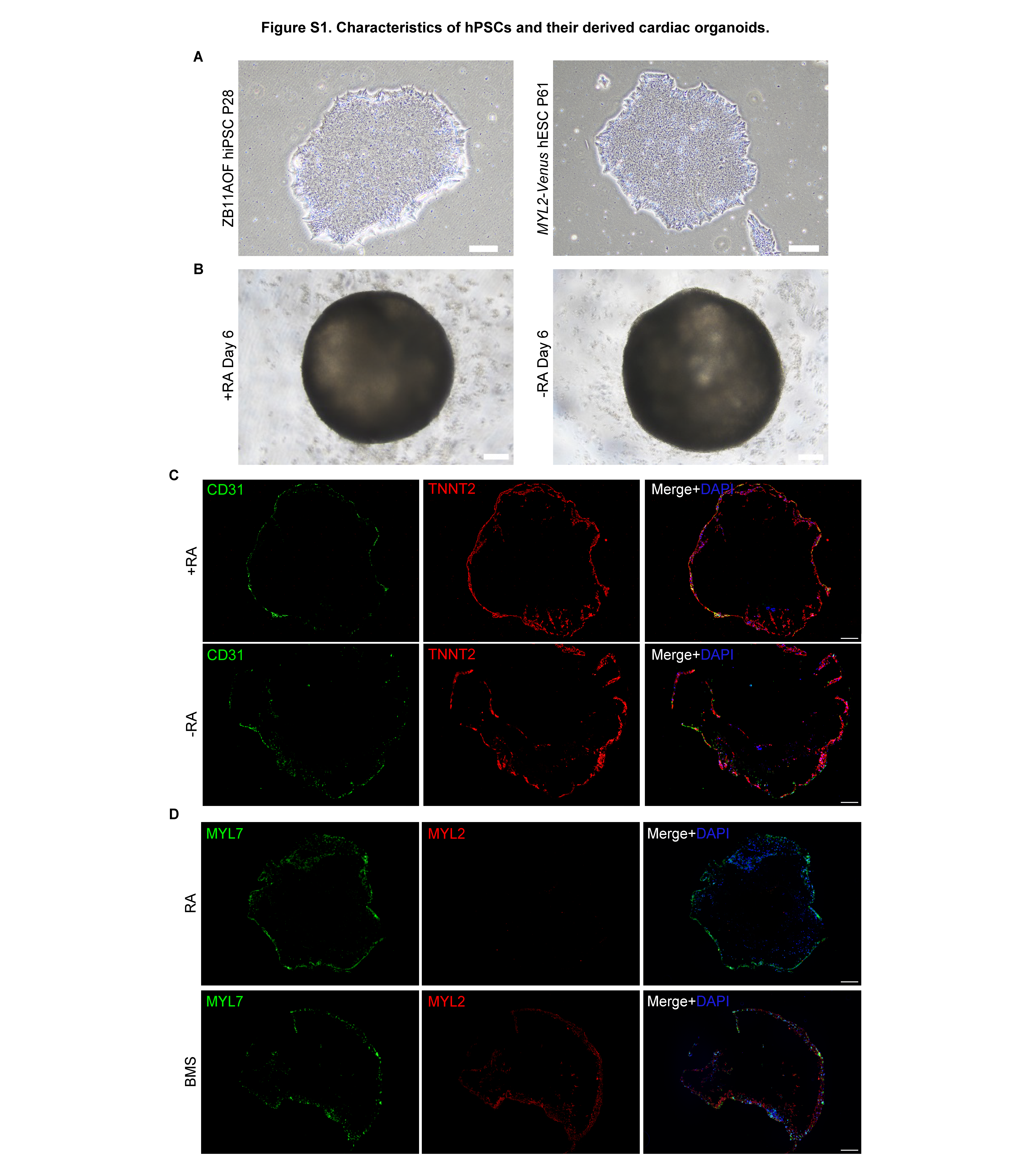

Supplement: Supplementary file 1 — Figure S1. Characteristics of hPSCs and their derived cardiac organoids. (A) Bright‐field images of ZB11AOF hiPSC and MYL2‐Venus hESC. Scale bars, 100 μm. (B) Bright‐field microscopy images of +RA and ‐RA treated cardiac organoids at Day 6. Scale bar, 200 μm. (C) Immunofluorescence staining of TNNT2 (red) and CD31 (green) for cardiac organoids fabricated in +RA and ‐RA treated groups on Day 8. Scale bars, 100 μm. (D) Immunofluorescence staining of MYL2 (red) and MYL7 (green) for cardiac organoids fabricated in RA and BMS treated groups on Day 8. Scale bars, 100 μm. +RA and RA indicate adding retinoic acid, −RA indicates removing retinoic acid, BMS indicates BMS493. [file CPR-58-e13762-s003.tif]

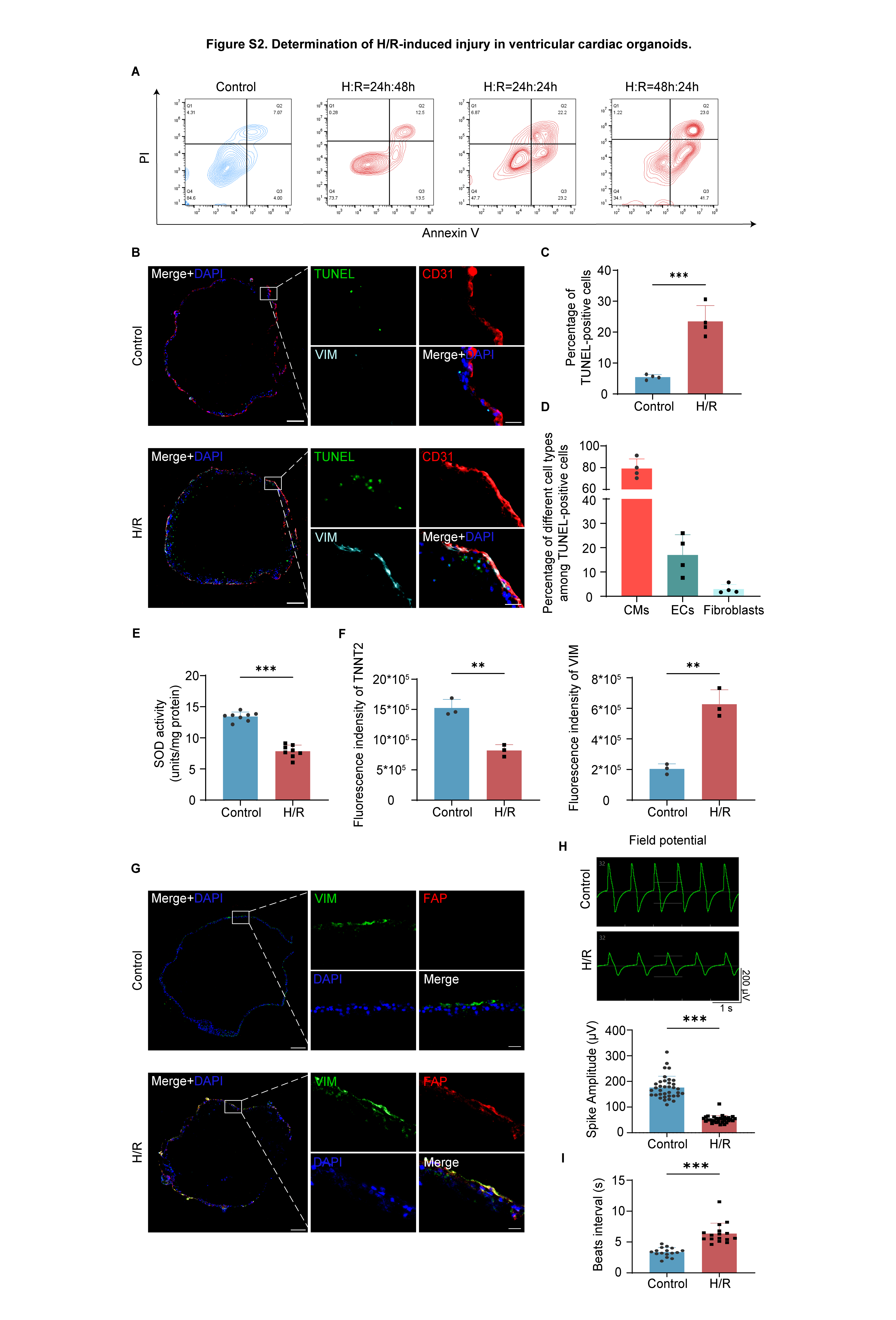

Supplement: Supplementary file 2 — Figure S2. Determination of H/R‐induced injury in ventricular cardiac organoids. (A) Proportions of early and late apoptotic cells for different time ratio of hypoxia and reoxygenation by flow cytometry analysis. (B) Distribution of CD31 (red), VIM (cyan), TUNEL (green) and DAPI (blue) in control and H/R groups. Scale bars (left), 200 μm. Scale bars (right), 20 μm. (C) Percentage of TUNEL‐positive cells, n = 4 independent replicates. (D) Percentage of different cell types among TUNEL‐positive cells, n = 4 independent replicates. (E) SOD activity in control and H/R groups, n = 8 independent replicates per group. (F) Flurorescence intensity of TNNT2 and VIM in Figure 2G, n = 3 images per group. (G) Distribution of FAP (red), VIM (green) and DAPI (blue) in control and H/R groups. Scale bars (left), 200 μm. Scale bars (right), 20 μm. (H) MEA‐measured field potential and spike amplitude values (n = 36 electrodes) in control and H/R groups. (I) Interval time (s) between every beating, n = 16 organoids. H/R indicates hypoxia/reoxygenation and VIM indicates Vimentin. Bar and dot plot graphs show mean ± SD. Statistical significance was assessed by unpaired t‐test (**p < 0.01 and ***p < 0.001). [file CPR-58-e13762-s001.tif]

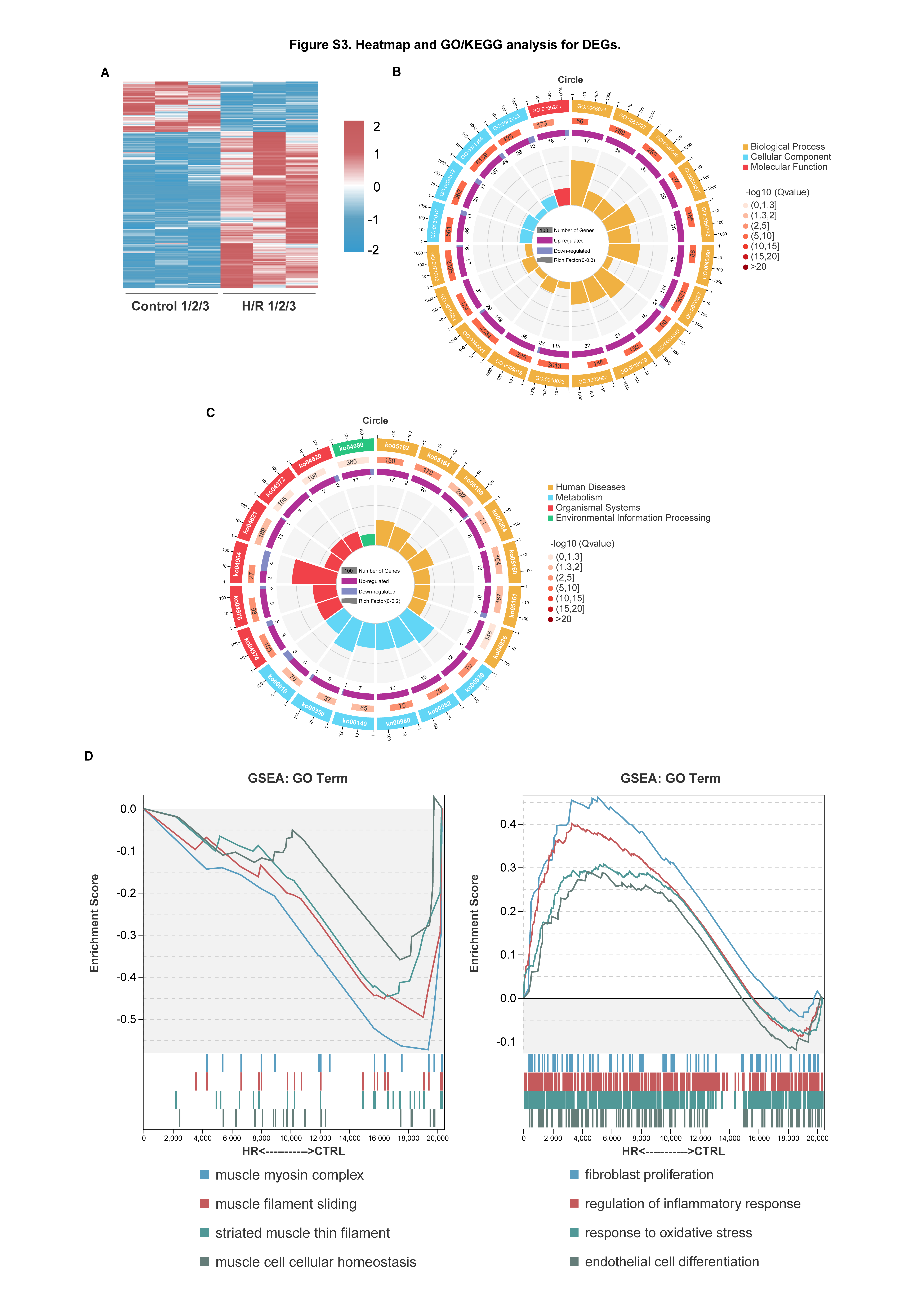

Supplement: Supplementary file 3 — Figure S3. Heatmap and GO/KEGG analysis for DEGs. (A) Heatmap displaying the expression patterns of differentially expressed genes. (B) GO enrichment analysis based on DEGs between Control and H/R group. (C) KEGG enrichment analysis based on DEGs between Control and H/R group. (D) GSEA plot of pathways related to CMs structural disruption, ECs stress, fibroblasts activation, and response with stress and inflammation from the GO enrichment analysis. H/R indicates hypoxia/reoxygenation. [file CPR-58-e13762-s006.tif]

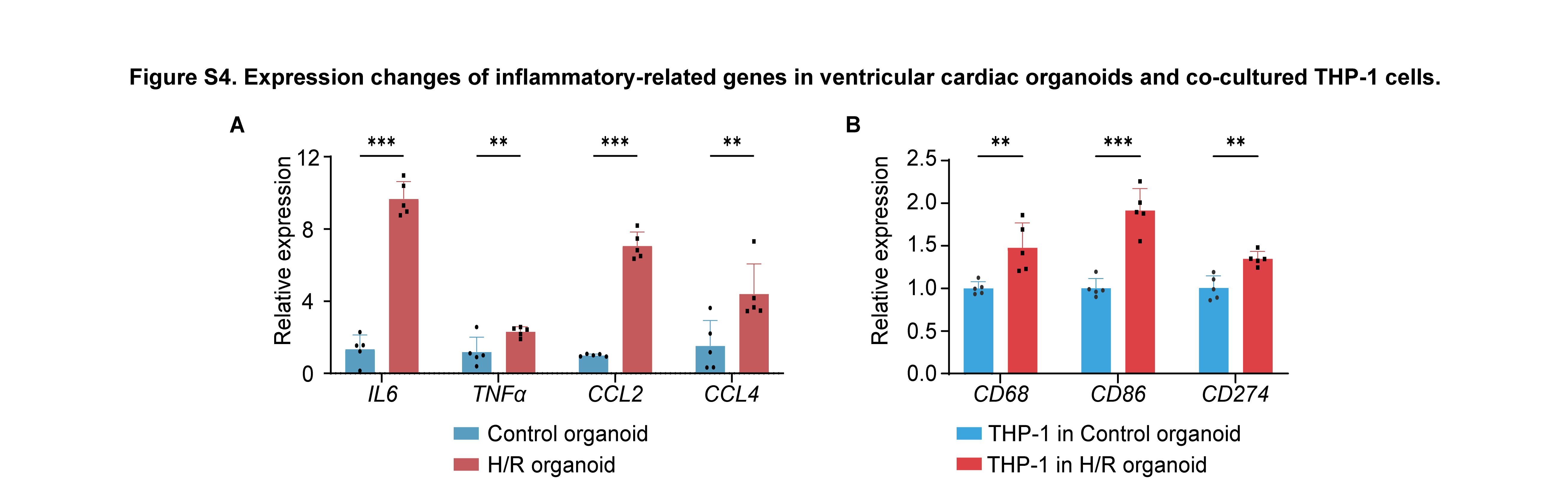

Supplement: Supplementary file 4 — Figure S4. Expression changes of inflammatory‐related genes in ventricular cardiac organoids and co‐cultured THP‐1 cells. (A) mRNA expression levels of pro‐inflammatory factors in control and H/R groups of ventricular cardiac organoids, n = 5. (B) mRNA expression levels of M1 macrophage polarization markers in THP‐1 cells co‐cultured with control and H/R‐induced ventricular cardiac organoids, n = 5. H/R indicates hypoxia/reoxygenation. Bar and dot plot graphs show mean ± SD. Statistical significance was assessed by unpaired t‐test and one‐way ANOVA (**p < 0.01, ***p < 0.001, ns indicates not significant). [file CPR-58-e13762-s005.tif]
