# Supplementary material for: Modelling myocardial ischemia/reperfusion injury with inflammatory response in human ventricular cardiac organoids
Source: Cell Prolif. 2024 Oct 8;58(3):e13762. doi: 10.1111/cpr.13762 (PMC11882745; doi:10.1111/cpr.13762)
Supplement: Supplementary file 8 — Table S1. Primer sets used for qPCR experiments. [file CPR-58-e13762-s008.docx]

**Supplemental information**

**Modelling myocardial ischemia/reperfusion injury**

**with inflammatory response in**

**human ventricular cardiac organoids**

**Laihai Zhang^1,2^, Yun Jiang^3^, Wenwen Jia^4^, Wenjun Le^3^, Jie Liu^1^, Peng Zhang^3,5^, Huangtian Yang^1,3,5,*^, Zhongmin Liu^1,2,3,6,*^ and Yang Liu^1,3,*^**

**^1^** Shanghai Heart Failure Research Center, Shanghai East Hospital, School of Medicine, Tongji University, Shanghai 200092, China.

**^2^** Department of Cardiovascular Surgery, Shanghai East Hospital, School of Medicine, Tongji University, Shanghai 200092, China.

**^3^** Institute for Regenerative Medicine, Shanghai East Hospital, School of Medicine, Tongji University, Shanghai 200092, China.

**^4^** National Stem Cell Translational Resource Center, Shanghai East Hospital, School of Life Sciences and Technology, Tongji University, Shanghai, 200092, China.

**^5^** Laboratory of Molecular Cardiology, Shanghai Institute of Nutrition and Health, University of Chinese Academy of Sciences (CAS), CAS, Shanghai, 200031, China.

**^6^** Shanghai Institute of Stem Cell Research and Clinical Translation, Shanghai, 200120, China.

* Correspondence: liuyang4571@tongji.edu.cn (Y.L.), liu.zhongmin@tongji.edu.cn (Z.L.), htyang@sinh.ac.cn (H.Y.)

**Running title:**

Modelling myocardial I/R injury in human ventricular cardiac organoids

**Supplemental information titles and legends**

**Figure S1. Characteristics of hPSCs and their derived cardiac organoids**

(**A**) Bright-field images of ZB11AOF hiPSC and *MYL2-Venus* hESC. Scale bars, 100 μm. (**B**) Bright-field microscopy images of +RA and -RA treated cardiac organoids at Day 6. Scale bar, 200 μm. (**C**) Immunofluorescence staining of TNNT2 (red) and CD31 (green) for cardiac organoids fabricated in +RA and -RA treated groups on Day 8. Scale bars, 100 μm. (**D**) Immunofluorescence staining of MYL2 (red) and MYL7 (green) for cardiac organoids fabricated in RA and BMS treated groups on Day 8. Scale bars, 100 μm. +RA and RA indicate adding retinoic acid, -RA indicates removing retinoic acid, BMS indicates BMS493.

**Figure S2. Determination of H/R-induced injury in ventricular cardiac organoids**

(**A**) Proportions of early and late apoptotic cells for different time ratio of hypoxia and reoxygenation by flow cytometry analysis. (**B**) Distribution of CD31 (red), VIM (cyan), TUNEL (green) and DAPI (blue) in control and H/R groups. Scale bars (left), 200 μm. Scale bars (right), 20 μm. (**C**) Percentage of TUNEL-positive cells, n=4 independent replicates. (**D**) Percentage of different cell types among TUNEL-positive cells, n=4 independent replicates. (**E**) SOD activity in control and H/R groups, n=8 independent replicates per group. (**F**) Flurorescence intensity of TNNT2 and VIM in Figure 2G, n=3 images per group. (**G**) Distribution of FAP (red), VIM (green) and DAPI (blue) in control and H/R groups. Scale bars (left), 200 μm. Scale bars (right), 20 μm. (**H**) MEA-measured field potential and Spike Amplitude values (n=36 electrodes) in control and H/R groups. (**I**) Interval time (s) between every beating, n=16 organoids. H/R indicates hypoxia/reoxygenation and VIM indicates Vimentin. Bar and dot plot graphs show mean ± SD. Statistical significance was assessed by unpaired t-test (**p < 0.01 and ***p < 0.001).

**Figure S3. Heatmap and GO/KEGG analysis for DEGs**

(**A**) Heatmap displaying the expression patterns of differentially expressed genes. (**B**) GO enrichment analysis based on DEGs between Control and H/R group. (**C**) KEGG enrichment analysis based on DEGs between Control and H/R group. (**D**) GSEA plot of pathways related to CMs structural disruption, ECs stress, fibroblasts activation, and response with stress and inflammation from the GO enrichment analysis. H/R indicates hypoxia/reoxygenation.

**Figure S4. Expression changes of inflammatory-related genes in ventricular cardiac organoids and co-cultured THP-1 cells**

(**A**) mRNA expression levels of pro-inflammatory factors in control and H/R groups of ventricular cardiac organoids, n=5. (**B**) mRNA expression levels of M1 macrophage polarization markers in THP-1 cells co-cultured with control and H/R-induced ventricular cardiac organoids, n=5. H/R indicates hypoxia/reoxygenation. Bar and dot plot graphs show mean ± SD. Statistical significance was assessed by unpaired t-test and one-way ANOVA (**p < 0.01, ***p < 0.001, ns indicates not significant).

**Table S1. Primer sets used for qPCR experiments**

| Genes | Sequences (5’-3’) | |
| --- | --- | --- |
| *GAPDH* | Forward | TGCACCACCAACTGCTTAGC |
|  | Reverse | GGCATGGACTGTGGTCATGAG |
| *ACTB* | Forward | TTCCTTCCTGGGCATGGAGT |
|  | Reverse | TCTTCATTGTGCTGGGTGCC |
| *TNNT2* | Forward | ATGATGCATTTTGGGGGTTA |
|  | Reverse | CAGCACCTTCCTCCTCTCAG |
| *MYL2* | Forward | TTGGGCGAGTGAACGTGAAAA |
|  | Reverse | CCGAACGTAATCAGCCTTCAG |
| *MYL7* | Forward | TCAGCTGTATCGACCAGAATCG |
|  | Reverse | AAGACGGTGAAGTTGATGGG |
| *IRX4* | Forward | GGCTCCCCAGTTCTTGATGG |
|  | Reverse | TAGACCGGGCAGTAGACCG |
| *IL6* | Forward | CCTGAACCTTCCAAAGATGGC |
|  | Reverse | TTCACCAGGCAAGTCTCCTCA |
| *TNFα* | Forward | CCTCTCTCTAATCAGCCCTCTG |
|  | Reverse | GAGGACCTGGGAGTAGATGAG |
| *CCL2* | Forward | CAGCCAGATGCAATCAATGC |
|  | Reverse | GGAATCCTGAACCCACTTCT |
| *CCL4* | Forward | CTGTGCTGATCCCAGTGAATC |
|  | Reverse | TCAGTTCAGTTCCAGGTCATACA |
| *CD68* | Forward | GGAAATGCCACGGTTCATCCA |
|  | Reverse | TGGGGTTCAGTACAGAGATGC |
| *CD86* | Forward | CTGCTCATCTATACACGGTTACC |
|  | Reverse | GGAAACGTCGTACAGTTCTGTG |
| *CD274* | Forward | TGGCATTTGCTGAACGCATTT |
|  | Reverse | TGCAGCCAGGTCTAATTGTTTT |
| *MX1* | Forward | GGTGGTCCCCAGTAATGTGG |
|  | Reverse | CGTCAAGATTCCGATGGTCCT |
| *OAS2* | Forward | CTCAGAAGCTGGGTTGGTTTAT |
|  | Reverse | ACCATCTCGTCGATCAGTGTC |
| *IRF9* | Forward | GCCCTACAAGGTGTATCAGTTG |
|  | Reverse | TGCTGTCGCTTTGATGGTACT |
| *ISG15* | Forward | TGGACAAATGCGACGAACCTC |
|  | Reverse | TCAGCCGTACCTCGTAGGTG |
| *IFIT1* | Forward | GCGCTGGGTATGCGATCTC |
|  | Reverse | CAGCCTGCCTTAGGGGAAG |
| *STAT1* | Forward | ATCAGGCTCAGTCGGGGAATA |
|  | Reverse | TGGTCTCGTGTTCTCTGTTCT |
| *OAS1* | Forward | TGTCCAAGGTGGTAAAGGGTG |
|  | Reverse | CCGGCGATTTAACTGATCCTG |
| *OAS3* | Forward | GCTTCAAGAGCTATGTGGACC |
|  | Reverse | GGAAACGTGAGTCTCAGACCA |
| *DDX60* | Forward | CAGCTCCAATGAAATGGTGCC |
|  | Reverse | CTCAGGGGTTTATGAGAATGCC |
| *PARP9* | Forward | TGCAATGGTCGTGAACAACCT |
|  | Reverse | CAACTGGGACCGTTGAAACTG |
| *PARP14* | Forward | TGTTAGTGGAGAACATAAGTGGC |
|  | Reverse | TGAATGGTGCTTGGTACAATCAT |
| *IFI6* | Forward | GGTCTGCGATCCTGAATGGG |
|  | Reverse | TCACTATCGAGATACTTGTGGGT |
| *IFI44L* | Forward | AGCCGTCAGGGATGTACTATAAC |
|  | Reverse | AGGGAATCATTTGGCTCTGTAGA |
| *IFI44* | Forward | GGTGGGCACTAATACAACTGG |
|  | Reverse | CACACAGAATAAACGGCAGGTA |
| *IFI27* | Forward | TGCTCTCACCTCATCAGCAGT |
|  | Reverse | CACAACTCCTCCAATCACAACT |
| *NR2F2* | Forward  Reverse | AACCAGCCGACGAGATTCG  CCCGGATGAGGGTTTCGATG |
| *NPPA* | Forward | CAACGCAGACCTGATGGATTT |
|  | Reverse | AGCCCCCGCTTCTTCATTC |

**Video S1.** Human chambered cardiac organoids induced by RA show spontaneous beating, related to Figure 1

**Video S2.** Human ventricular cardiac organoids induced by BMS493 show spontaneous beating, related to Figure 1

**Video S3.** H/R-induced human ventricular cardiac organoids show weakened beating property, related to Figure 2
